# A small molecule high throughput screening platform to profile conformational properties of nascent, ribosome-bound proteins

**DOI:** 10.1038/s41598-022-06456-5

**Published:** 2022-02-15

**Authors:** Hideki Shishido, Jae Seok Yoon, William R. Skach

**Affiliations:** 1 CFFT Lab, Cystic Fibrosis Foundation, 44 Hartwell Ave, Lexington, MA 02421 USA; 2 Cystic Fibrosis Foundation, 4550 Montgomery Ave., Suite 1100N, Bethesda, MD 20814 USA; 3 Present Address: Generate Biomedicines, Inc., 26 Landsdowne St, Cambridge, MA 02139 USA

**Keywords:** Protein folding, High-throughput screening, Protein folding

## Abstract

Genetic mutations cause a wide spectrum of human disease by disrupting protein folding, both during and after synthesis. Transient de-novo folding intermediates therefore represent potential drug targets for pharmacological correction of protein folding disorders. Here we develop a FRET-based high-throughput screening (HTS) assay in 1,536-well format capable of identifying small molecules that interact with nascent polypeptides and correct genetic, cotranslational folding defects. Ribosome nascent chain complexes (RNCs) containing donor and acceptor fluorophores were isolated from cell free translation reactions, immobilized on Nickel-NTA/IDA beads, and imaged by high-content microscopy. Quantitative FRET measurements obtained from as little as 0.4 attomole of protein/bead enabled rapid assessment of conformational changes with a high degree of reproducibility. Using this assay, we performed a pilot screen of ~ 50,000 small molecules to identify compounds that interact with RNCs containing the first nucleotide-binding domain (NBD1) of the cystic fibrosis transmembrane conductance regulator (CFTR) harboring a disease-causing mutation (A455E). Screen results yielded 133 primary hits and 1 validated hit that normalized FRET values of the mutant nascent peptide. This system provides a scalable, tractable, structure-based discovery platform for screening small molecules that bind to or impact the folding of protein substrates that are not amenable to traditional biochemical analyses.

## Introduction

In cells, nascent proteins begin to acquire three dimensional tertiary structure cotranslationally in a complex cellular environment as the nascent polypeptide emerges from the ribosome exit tunnel^1–5^. De novo protein folding can be influenced by biosynthetic machinery^3,4^ including the presence of the adjacent ribosome^2–4^, translation elongation rate^2–4,6,7^, interactions with cellular chaperones^3,4,8,9^, and molecular crowding^10–12^. Events that occur during synthesis can also influence folding efficiency as well as functional properties of the final structure^13,14^. When folding is disrupted either by inherited or acquired mutations or perturbations of the cellular environment, resultant misfolded proteins can cause a diverse array of clinical disorders through loss of function, gain of toxic function, or cellular mislocalization and degradation by the ubiquitin proteasome system (UPS)^15–18^.

Cystic fibrosis (CF) is a prototypical protein folding disorder caused by mutations in the Cystic Fibrosis Transmembrane conductance Regulator (CFTR), an ABC transporter containing two six-spanning transmembrane domains (TMD1 and TMD2), two cytosolic nucleotide binding domains (NBD1 and NBD2), and an unstructured regulatory domain (R). CFTR functions as a PKA-regulated and ATP-gated chloride channel in the apical membrane of epithelial tissues where it controls salt and water movement^19^. More than 1,700 genetic variants have been reported for CFTR (www.genet.sickkids.on.ca/cftr), and it is estimated that several hundred variants cause CF by disrupting CFTR folding through various mechanisms. For example, F508del, which eliminates a phenylalanine at residue 508, decreases thermal stability of NBD1^20,21^ and destabilizes an intramolecular interface between NBD1 and the 4th intracellular loop of TMD2^21–23^. NBD1 folding is particularly susceptible to disease causing mutations due to its complex folding pathway, in which cellular biosynthetic machinery is required to facilitate formation of a parallel 4-stranded hydrophobic β-sheet within the core of the domain^1,2,24^. Recently, we showed that certain mutations in NBD1 can also alter the cotranslational folding pathway and thereby influence CFTR trafficking and stability^24^.

High-throughput screening (HTS) strategies identifying novel pharmacological chaperones for protein misfolding disorders, have typically relied on cellular assays such as measurements of the cell surface expression or function of target proteins^25–27^. For CF, phenotypic cell-based HTS assays have been widely used to screen for corrector molecules^28,29^ based on CFTR function at the plasma membrane^30,31^ or the trafficking of mutant CFTR. Purified protein domains have also been used in thermal shift assays^32,33^ to identify molecules that bind NBD1 directly. To date, HTS efforts have largely focused on F508del and have identified numerous corrector molecules^34^. These extensive efforts to correct F508del CFTR folding in cells, using small molecules, have led to recent FDA approval of three combination drugs: Lumacaftor (VX-809) + Ivacaftor in 2015, Tezacaftor (VX-661) + Ivacaftor in 2018, and Elexacaftor (VX-445) + Tezacaftor + Ivacaftor in 2019^35–38^. Despite these advances, however, not all CFTR missense mutations respond to existing modulator drugs^39^. In addition, compounds must be present during CFTR synthesis to maximally stimulate CFTR folding^36,40,41^. This has suggested that some CFTR correctors may act on one or more transient biosynthetic intermediates^42^ and that understanding cotranslational folding pathways may provide a potential novel approach for developing new treatment strategies^24^.

**Figure 1:**
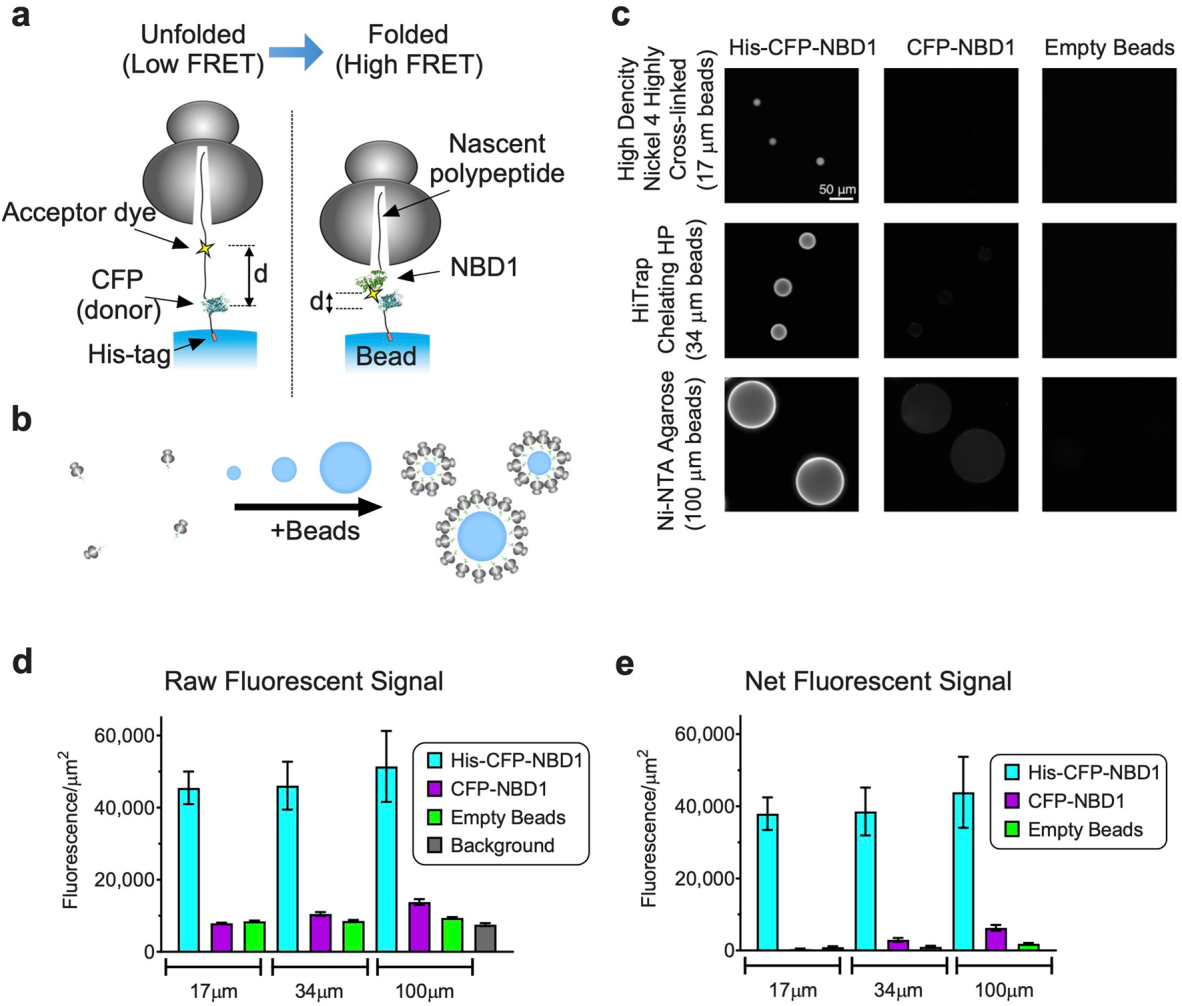
Fluorescent detection of immobilized RNCs. (**a**) Cartoon depicting solid-support FRET assay using translationally incorporated fluorophores to detect structural transitions of ribosome-attached nascent polypeptides immobilized on solid-support surface via His-tag. (**b**) Schematic showing increase in RNC concentration on surface of beads. (**c**) Raw images of beads containing RNC with His_10_-CFP-NBD1. Equivalent amount of RNCs were incubated with 2 × 10^5^ of 17 µm beads, 5 × 10^4^ of 34 µm beads, and 6 × 10^3^ of 100 µm beads as described in “Methods” section. Scale bar, 50 µm. (**d**) Mean fluorescence intensity/µm^2^ was obtained from images (mean ± SD, n = 12–99 beads). Mean fluorescence intensities of beads containing His-CFP-NBD1 RNCs and background signal intensity (buffer) were used to calculate signal to background ratios. (**e**) Net fluorescence signal intensity/µm^2^ was determined by subtracting background (buffer) (mean ± SD).

A powerful method to study transient biosynthetic folding intermediates utilizes stable, Ribosome-bound Nascent Chain complexes (RNCs) derived from in vitro-translated truncated RNA transcripts^1,2,43,44^. In this system it is possible to quantitatively insert a donor fluorophore, Cyan Fluorescent Protein (CFP) at the N-terminus via a chimeric fusion, and a small acceptor dye (7-nitrobenz-2-oxa-1,3-diazole) at an engineered stop (TAG) codon within NBD1 using a synthetic aminoacylated suppressor tRNA (εN-7-nitrobenz-2-oxa-1,3-diazol (εNBD)-[^14^C]Lys-tRNA^amb^)^1,2,44^. Ribosomes that readthrough the stop codon, incorporate the acceptor dye and are arrested at the end of the truncated transcript at a defined stage of synthesis. Resultant polypeptides remain stably bound to the ribosome via a covalent peptidyl-tRNA bond (CFP-NBD1 RNCs), and are thus captured in the context of native biosynthetic machinery^44^. By adjusting translation conditions appropriately, donor and acceptor probes can be incorporated in near 1:1 stoichiometry, and RNC concentration can be precisely determined by incorporating a [^14^C] isotope into polypeptides during readthrough of the stop-codon (see “Methods”). It is therefore possible to simultaneously quantify nascent chain concentration and fluorescence intensity of donor and acceptor probes to determine Fluorescence Resonance Energy Transfer (FRET) at each truncation site as the nascent polypeptide transitions from an elongated, unfolded conformation (low FRET) to a more compact, folded conformation (high FRET)^1,2,24,45^ (Fig. 1a). Using this system, we previously showed that: (i) NBD1 acquires its structure cotranslationally through a distinct series of carefully choreographed folding events, (ii) CF-causing mutations located within NBD1 can disrupt the nascent polypeptide folding landscape, and (iii) genetic suppressor mutations that restore cotranslational folding can partially restore trafficking of full-length CFTR^1,2,24^. These findings suggest that nascent folding intermediates may play an important role in disease pathogenesis and thus provide potential targets for pharmacological correction.

While cell-free translation allows one to precisely control both nascent chain length and site of probe incorporation, FRET analysis is technically challenging due to the low concentration of RNCs (~ 1–5 nM) that can be isolated from in vitro translation reactions. We therefore employed a strategy to immobilize RNCs to increase the effective local concentration and enable quantitative fluorescence measurements via high-content microscopic imaging (Fig. 1b). This approach dramatically increases sensitivity, enabling us to detect approximately 0.4 attomole of RNCs per bead (2.4 × 10^5^ molecules), and allows ~ 20,000 FRET measurements to be made from a single scaled translation reaction. By implementing a series of technical refinements, we were able to establish this system for HTS in 1,536-well plate format with high degree of reproducibility, and performed a pilot screen using a ~ 50,000 small molecule compound library.

## Results

### Optimizing RNC binding and fluorescence detection

To improve sensitivity and scalability of fluorescence measurements, we systematically tested three immobilization strategies (streptavidin–biotin, His_10_-Ni–NTA/IDA, and myc (9E10) epitope antibody binding) using 12 commercially available solid supports (Supplementary Table S1). Ni–NTA/IDA binding was deemed most efficient, and three supports were identified with favorable binding efficiency, specificity, and optical characteristics: *High Density Nickel 4 Highly Cross-linked Superfine 17 µm* (17 µm beads), *HiTrap Chelating HP* charged with Nickel (34 µm beads), and *Ni–NTA agarose* (100 µm beads). Imaging was initially performed on an inverted Olympus IX71 and a Nikon Ti-E eclipse microscope, and subsequently adapted to a GE IN Cell Analyzer 2200 high content imaging system because of its superior performance and high throughput capabilities. Bead binding characteristics were optimized using His_10_-tagged and non-His-tagged CFP-NBD1 RNCs incubated with 2 × 10^5^ 17 µm beads, 5 × 10^4^ 34 µm beads, or 6 × 10^3^ 100 µm beads which provide similar aggregate binding surface area. All beads yielded satisfactory sixfold signal to background ratio (Fig. 1c–d). Net fluorescence signal was determined after flat field correction and subtraction of background (buffer) and nonspecific bead fluorescence (non-his-tagged constructs) (Fig. 1e). Net signal to noise (compared to non-His-tagged RNCs) was 103, 13, and 7 for 17 µm, 34 µm, and 100 µm beads, respectively. We also found that materials exhibited differences in variability in bead-to-bead fluorescence intensity, which impacted the reproducibility of individual FRET measurements. 17 µm beads were superior due to less variation in bead measurements and nonspecific binding compared to 34 µm and 100 µm beads (Fig. 1e and Supplementary Fig. S1). For these reasons, 17 µm beads (isolated within a narrow size range—16 µm to 23 µm—by gravity segmentation) were selected among the three Ni–NTA/IDA supports for further study.

**Figure 2:**
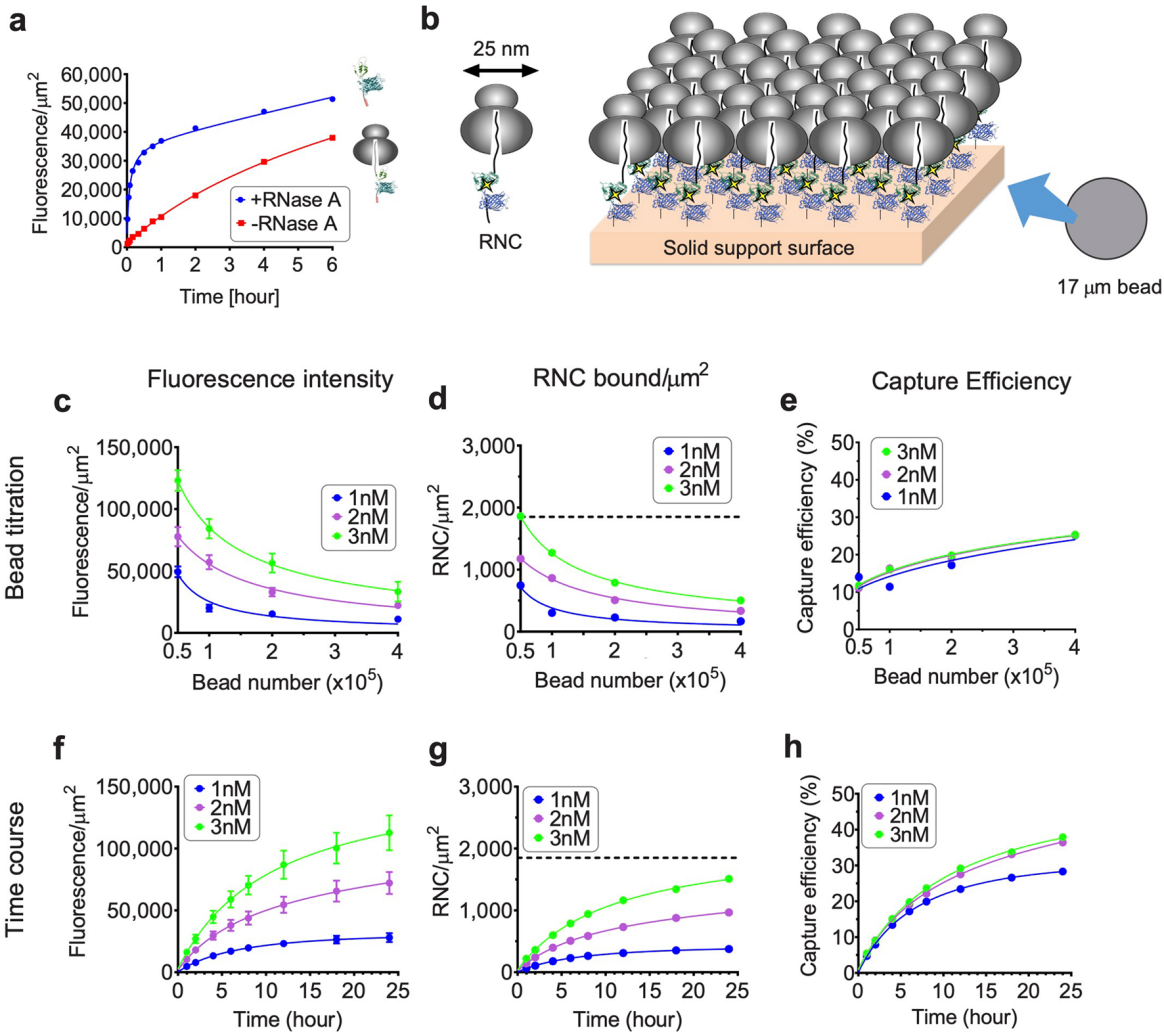
RNC capture is dependent on RNC concentration, binding time, and bead number. (**a**) Time course of bead binding for 2 × 10^5^ of 17 µm beads and 2 nM purified RNCs (His_10_-CFP-NBD1, donor only) ± RNase A treatment. (**b**) Cartoon depicting ribosome binding geometry on solid support surface assuming smooth surface. Diameter of ribosome is approximately 25 nm, yielding RNC saturation density of: $\eta$_h_(1 µm)^2^/($\pi$*r*^*2*^) = 1,850 ribosomes/µm^2^, where $\eta$_h_ is the coefficient of densest packing of circle in the plane, and *r* is radius of ribosome. (**c**–**e**) Effect of bead number on RNC binding. 1, 2, or 3 nM RNCs (His_10_-CFP-NBD1, donor only) were incubated with 0.5–4 × 10^5^ of 17 µm beads for 6 h. (**f**–**h**) Effect of incubation time on RNC binding. 1, 2, or 3 nM RNCs (His_10_-CFP-NBD1 donor only) incubated with 2 × 10^5^ of 17 µm beads for times indicated. Fluorescence intensity of 17 µm beads in panels **c** and **f** are shown in mean ± SD (n =  ~ 400 beads). Binding density in panels **d** and **g** was calculated using number of protein molecules bound per total calculated surface area of beads added. Dotted line in panels **d** and **g** indicates theoretical RNC saturation density as described in panel **b**. Capture efficiency in panels **e** and **h** was calculated by RNCs bound/RNCs in binding reaction.

RNC bead capture efficiency was evaluated by varying bead number and binding time (Fig. 2). Incubation times were chosen based on binding kinetics, which were much slower for RNCs than free polypeptides due to spatial and rotational constraints imposed by the attached ribosome (Fig. 2a,b). Total binding (fluorescent signal/µm^2^) was inversely proportional to bead number and increased by both RNC concentration and binding time (Fig. 2c,f). RNC surface density was calculated based on the amount of [^14^C]Lys-labeled nascent polypeptide bound per total surface area of beads using average bead diameter estimates (Fig. 2d,g). Surface density correlated well with fluorescence signal intensity. Bead capture efficiency (RNCs bound/RNCs in binding reaction) was time dependent but relatively unaffected by RNC concentration (Fig. 2e,h). Importantly, some bead binding conditions are outliers, which may be indications of inappropriate binding conditions. 34 µm, and 100 µm beads showed patterns similar to those of 17 µm beads (Supplementary Fig. S2, S3). However larger beads resulted in fewer usable measurements and greater variation in fluorescence intensity. From these results, 2 × 10^5^ 17 µm beads, 400 µl of 2 nM RNC and ≥ 6 h incubation time were chosen for further experiments as parameters that gave reasonable yield to use for HTS.

### Solid-support FRET using immobilized RNCs

**Figure 3:**
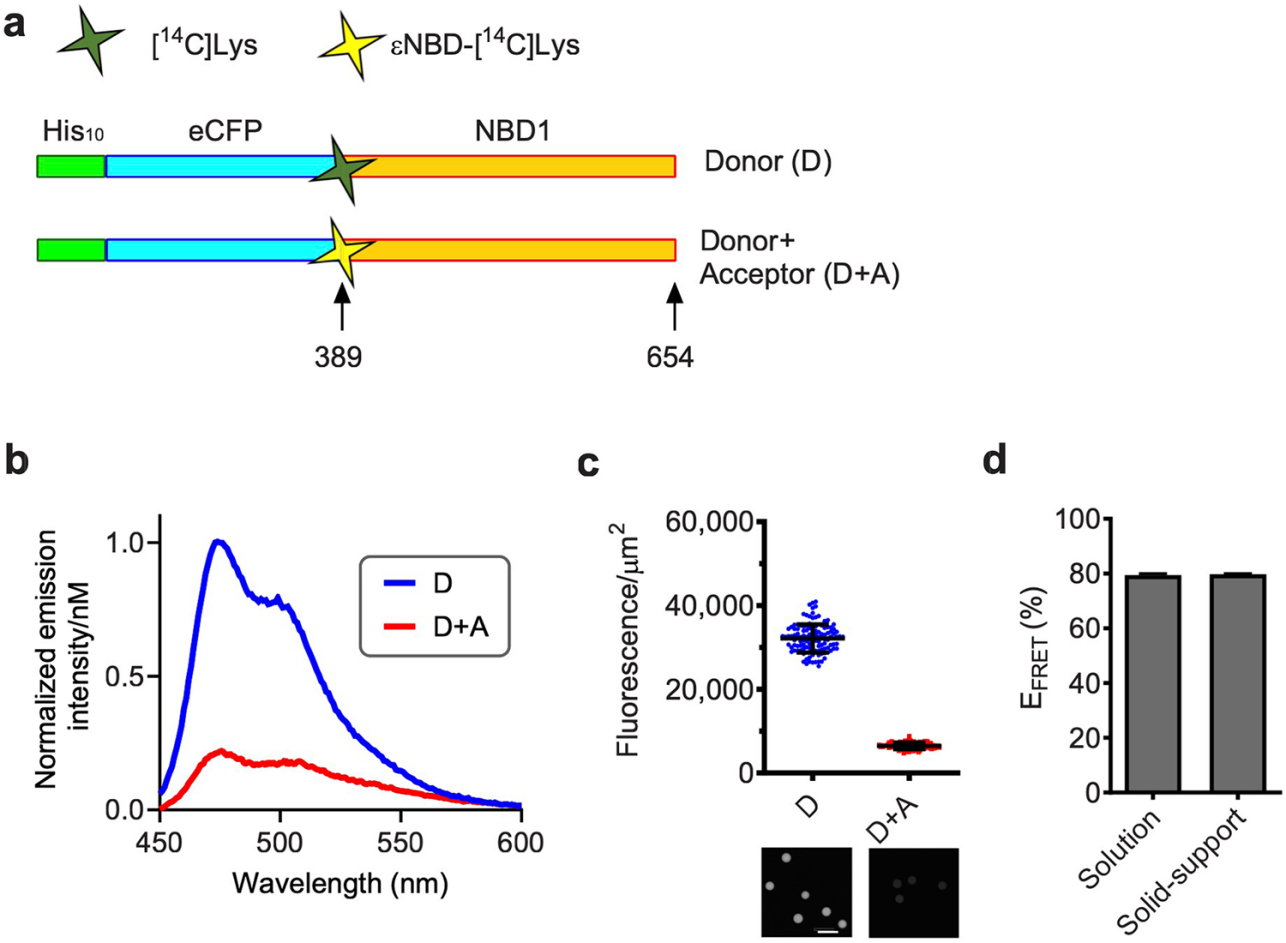
Solution FRET and Solid-support FRET of immobilized RNCs. (**a**) Schematic of His_10-_CFP-NBD1 construct showing approximate location of acceptor dye residue (389) and truncation site residue (654). (**b**) Graph showing CFP fluorescence emission spectra in solution in D and D + A samples. (**c**) Graph showing fluorescence intensity of 17 µm beads (mean ± SD, n = 118 or 108 for D or D + A beads, respectively). Raw images of beads containing D or D + A RNCs is shown below graph. Scale bar, 50 µm. (**d**) Graph showing Solution E_FRET_ and Solid-support E_FRET_. Data are mean ± SEM, n ≥ 3 independent experiments. Solution E_FRET_ was calculated from D, D + A, and blank samples based on CFP fluorescence intensity at λem = 475 nm (λex = 430 nm) as described in “Methods” section. Solid-support E_FRET_ was calculated from averaged bead fluorescence intensity in panel (**c**).

To determine whether substrate immobilization impacted nascent chain conformation, we compared steady state fluorescence measurements of RNCs in solution to measurements obtained from RNCs immobilized on bead surface. In each case, parallel, matched translation samples were used containing equal concentrations of nascent polypeptides that contained donor only (D), or donor + acceptor (D + A) fluorescent probes. For these experiments, the acceptor dye was incorporated at the CFP fusion site (residue 389 of CFTR) because FRET at this site depends only on the short tether distance to the CFP chromophore^1^ (Fig. 3a). FRET measurements were calculated as reported previously based on the decrease in donor fluorescence intensity due to the presence of the acceptor probe using the formula:

1 $${\text{E}}_{{{\text{FRET}}}} (\% ) = { 1} - {\text{F}}_{{{\text{DA}}}} /{\text{FD}}\times{1}00$$

(where E_FRET_ = FRET efficiency, F_DA_ = fluorescence intensities of donor in the presence of acceptor, and F_D_ = fluorescence in the presence of donor alone). Note that fluorescence of the acceptor is negligible under these conditions as described previously and therefore was not needed for determining FRET^1^. Solution-based measurements yielded a calculated E_FRET_ of 79 ± 0.5% (Fig. 3b), which is similar to previous reports and supported by theoretical distance estimates^1^. FRET values were calculated from beads containing immobilized RNCs prepared from matched translation reactions with D and D + A incorporated probes (~ 100 17 µm beads for D and D + A samples) based on mean F_D_ and F_DA_ per unit surface area (fluorescence units/μm^2^). Results of these calculations yielded E_FRET_ of 80 ± 0.2% in excellent agreement with solution-based measurements (Fig. 3c,d). Both solution and immobilized RNCs yielded FRET values that were somewhat higher than previously reported^1^, likely due to minor variations in experimental conditions and RNC quantitation. The correlation between solution-based measurements and solid-support measurements, indicate that fluorescence measurements and FRET values were relatively unaffected by conditions used for immobilization.

Previously^1,2^, we showed that during synthesis of the NBD1 N-terminal subdomain (CFTR residues 389 to 491), sequestration of C-terminal residues inside the ribosome retains the nascent chain in an unfolded conformation and that folding occurs abruptly as residues 500–550 exit the ribosome tunnel. To investigate whether RNC immobilization impacts the NBD1 folding status, nascent polypeptides containing an acceptor probe at residue 450 were truncated at residues 500 or 550, and E_FRET_ was determined before and after ribosome release (Supplementary Fig. S4a). Consistent with solution-based results (Supplementary Fig. S4b), immobilized ribosome-bound polypeptides truncated at residue 500 exhibited low E_FRET_ values (12 ± 4.2%), and E_FRET_ increased 2.5 fold following release from ribosome by RNase digestion (to 31 ± 3.3%) (Supplementary Fig. S4c). Similarly, at truncation 550, the N-terminal subdomain had already folded and little increase in FRET was observed upon ribosome release. Thus, RNC immobilization does not substantially impact NBD1 folding or affect the ability to detect changes in NBD1 conformation in ribosome bound and unbound states. In addition, RNC conformation was remarkably stable following storage at − 80 °C (Supplementary Fig. S5), thus enabling large batch preparation preformed RNCs for subsequent screening efforts. These results are also consistent with previous work demonstrating that ribosome-bound folding intermediates are remarkably stable^44^ in isolation buffer and provide a practical and scalable method to analyze transient folding intermediates.

### Detection of a cotranslational folding defect by solid-support FRET

**Figure 4:**
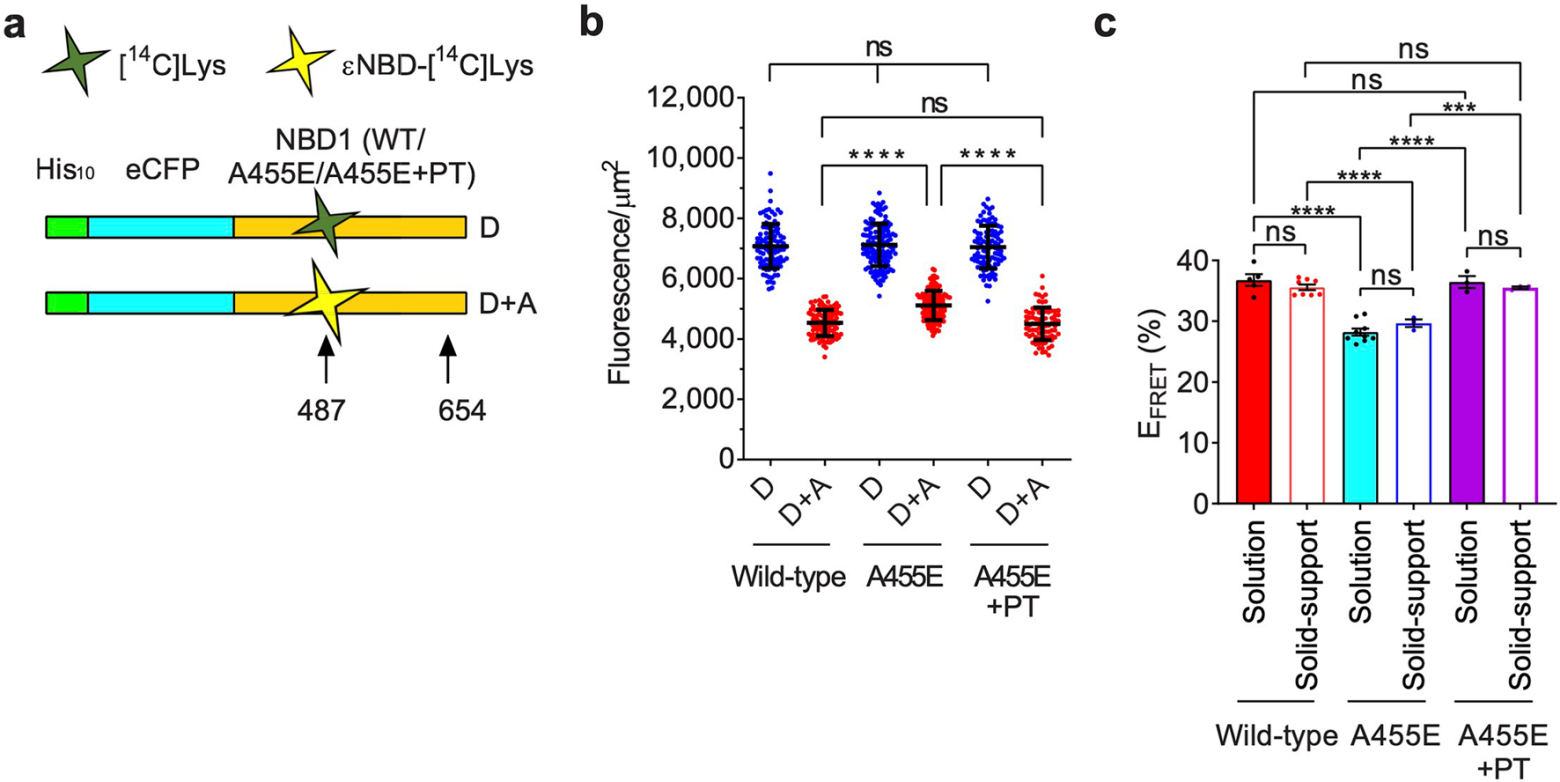
FRET analysis of wild-type or A455E NBD1 folding. (**a**) Schematic of His_10-_CFP-NBD1 (wild-type/A455E/A455E + PT) constructs showing approximate location of acceptor dye and truncation residues 487 and 654, respectively. (**b**) Graph showing fluorescence intensity of individual D or D + A beads for wild-type, A455E, or A455E + PT constructs (acceptor at 487 and truncation at 654) obtained from individual experiments in 384-well format (mean ± SD, n = 81–142 beads). (**c**) Graph showing E_FRET_ values obtained from individual experiments of solution or solid-support FRET assay in 384-well format for wild-type, A455E, or A455E + PT constructs indicated (mean ± SEM, n = 3–9 individual experiments). Two-tailed unpaired student’s t-test, ***p < 0.001, ****p < 0.0001, n.s. > 0.05.

Numerous mutations within NBD1 cause CF by disrupting NBD1/CFTR folding and/or intracellular trafficking^21–23,46^. Recently, we showed that A455E cotranslationally perturbs NBD1 α-helical subdomain folding with the most profound effect observed for nascent chains truncated at residue 654^24^. Consistent with solution-based measurements, A455E NBD1 also yielded significantly lower E_FRET_ (29 ± 0.1%) compared to wild-type (36 ± 0.5%) (mean ± SEM) in the solid-support FRET system (Fig. 4). Thus, immobilized RNCs reproduce mutation-induced deviations in the cotranslational folding pathway. In addition, we previously demonstrated that a combination of suppressor mutations (S492P and I539T (PT)) genetically corrected the cotranslational folding defect induced by A455E and partially restored folding of full-length CFTR trafficking in cells^24^. A suppressor mutation S492P is predicted to increase rigidity between the N-terminal and the α-helical subdomains^47^, and a well-studied suppressor mutation I539T^48^ is known to increase thermal stability of F508del NBD1. Like the PT, numerous suppressor mutations have been studied to understand CFTR/NBD1 folding defects, mechanism of action of corrector molecules, and used in some HTS systems^22,23,42,49^. As shown in Fig. 4c, the PT suppressor mutations also corrected the folding defect of A455E NBD1 observed by solid-support FRET^24^. These results suggested that immobilized A455E NBD1 cotranslational folding intermediates could be used to screen for small molecules that might interact with the nascent polypeptide and restore folding similar to the PT suppressor mutations.

Note that during our study, GE introduced a software update algorithm for flat field correction in the InCell system that reduced the values of reported fluorescence signal. However, this software change had no impact on FRET values (see Supplementary Fig. S6 and methods for details).

### Optimization of FRET detection in multi-well plates

Immobilized RNCs were initially tested in 96-well and 384-well plates by dispensing 1,000 beads in 200 µl/well or 500 beads in 50 µl/well, respectively, containing His_10_-CFP-NBD1 RNCs (acceptor probe at residue 450 and truncation at residue 550) into 48 wells (24 wells each for D and DA beads). Note that wild-type constructs were used for 96-well and 384-well plate assays as these trials were performed prior to the study of A455E cotranslational folding defect^24^. Four images were taken of each well, and approximately 30 beads were selected per well (after eliminating unfocused beads and artifacts) to quantify mean fluorescence intensity (see “Methods” section for details). Each matched pair of wells (D and D + A) therefore yielded a single FRET measurement (Supplementary Fig. S7). While 96-well and 384-well plate formats yielded nearly the same E_FRET_ values (50.6 ± 2.0%, and 50.3% ± 1.4%, respectively), the 384-well format showed less variation. Bead titration revealed that variation in fluorescence signal and E_FRET_ values improved as the number of images (9 per well) and beads (> 400/well) were increased in the 384-well plate format (Supplementary Fig. S8). Among known ABC transporters, NBD1 contains a unique unstructured regulatory region (RI: residues 405–436) of uncertain function. When deleted (∆RI), maturation, stability, and function of wild-type CFTR are improved and F508del CFTR function and trafficking in cells are partially restored^50,51^. RI deletion also improves solubility and thermostability of the isolated NBD1 domain^20,51,52^ through the stabilization of the α-subdomain and α/β-core. We therefore compared the efficiency of wild-type and ∆RI NBD1 folding in 384 well format. E_FRET_ values were 51.7 ± 0.7% for wild-type, and 61.6% ± 0.6% for ΔRI (Supplementary Fig. S9). Comparison of E_FRET_ values yielded 0.70 of estimated Z’ factor^53^, consistent with robust assay reproducibility. Importantly, ≥ 8% FRET difference between negative and positive controls was needed to obtain Z’ factor > 0.5 based on this result. For practical purposes, wild-type and ΔRI RNCs were used as negative and positive controls for the Z’ factor estimation.

**Figure 5:**
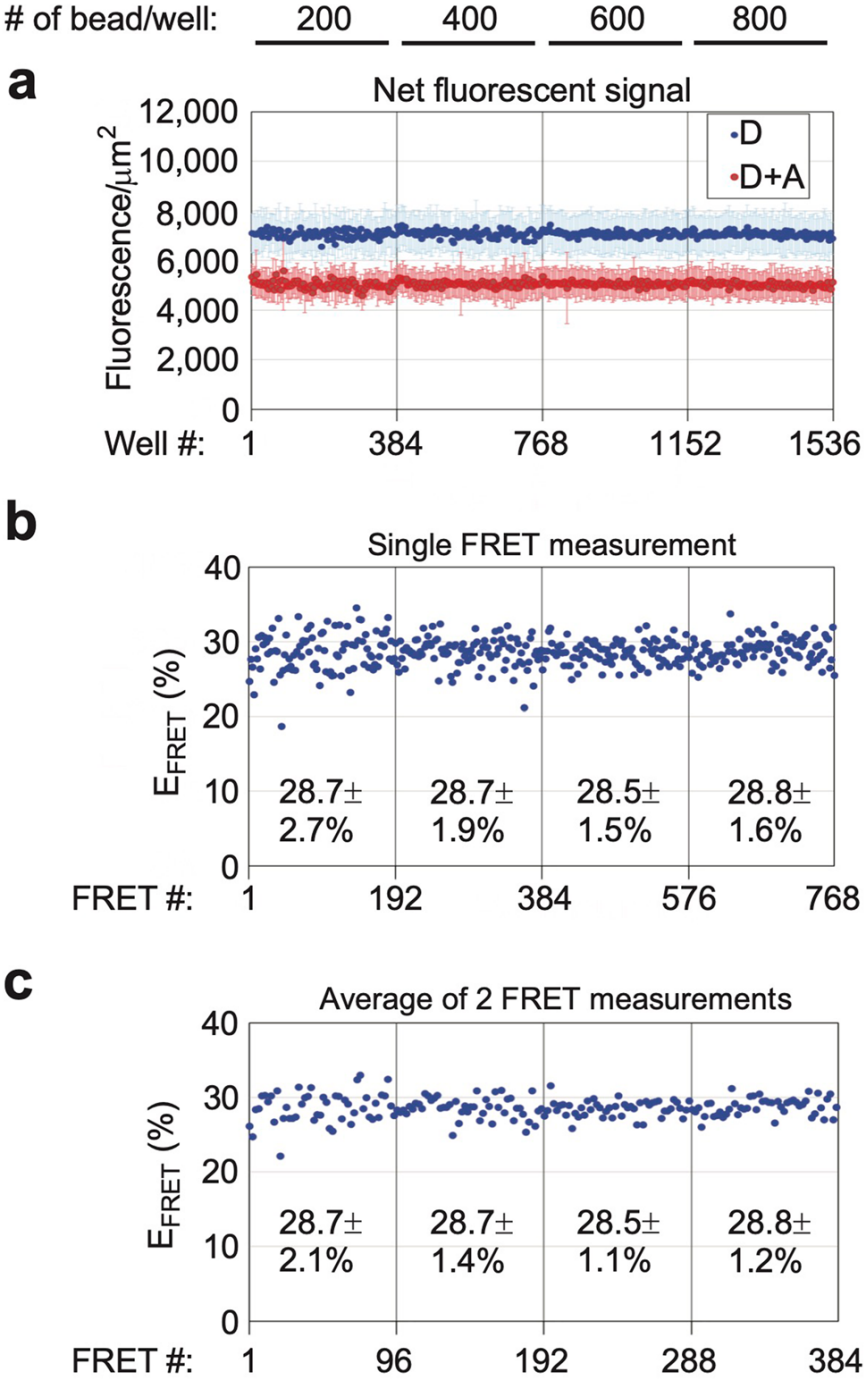
Bead titration in 1,536-well format. (**a**) Individual dots show mean fluorescence intensity ± SD for D and D + A samples bound to 17 µm beads in matched paired wells of a 1,536-well plate. Number of beads in each well is indicated at top. (1-image/well = 19% of total well area). (**b** & **c**) Individual E_FRET_ values calculated from single D and D + A pair of wells (**b**), or average of two D and D + A pairs (**c**). Mean E_FRET_ ± SD values were calculated for each bead binding condition (n = 192 single D and D + A pair or 96 average of two D and D + A pairs in panels **b** or **c**, respectively).

To improve scalability, we also evaluated a 1,536-well plate format: 2 nM RNCs containing A455E NBD1 (acceptor probe at residue 487, and truncation at residue 654) were incubated with 1 × 10^6^ 17 µm beads in 800 µL binding buffer for over-night (16 h). Reproducibility improved as the number of bead/well was increased to 600 (Fig. 5). Moreover, the average of two adjacent FRET measurements improved reproducibility of E_FRET_ value relative to individual FRET measurements (Fig. 5c). Given the small well area E_FRET_ values required only 1-image/well, substantially reducing the length of imaging time to 38 min for 768 FRET measurements versus 77 min for 192 FRET measurements in 384-well format (9-images/well). To account for imaging multiple plates over several hours, we showed that prolonged incubation at room temperature did not affect RNC stability in dilution buffer (Supplementary Fig. S10). Thus, multiple plates could be prepared and stored at room temperature for up to 24 h until imaging with little impact on data quality.

### FRET-based high throughput screening

**Figure 6:**
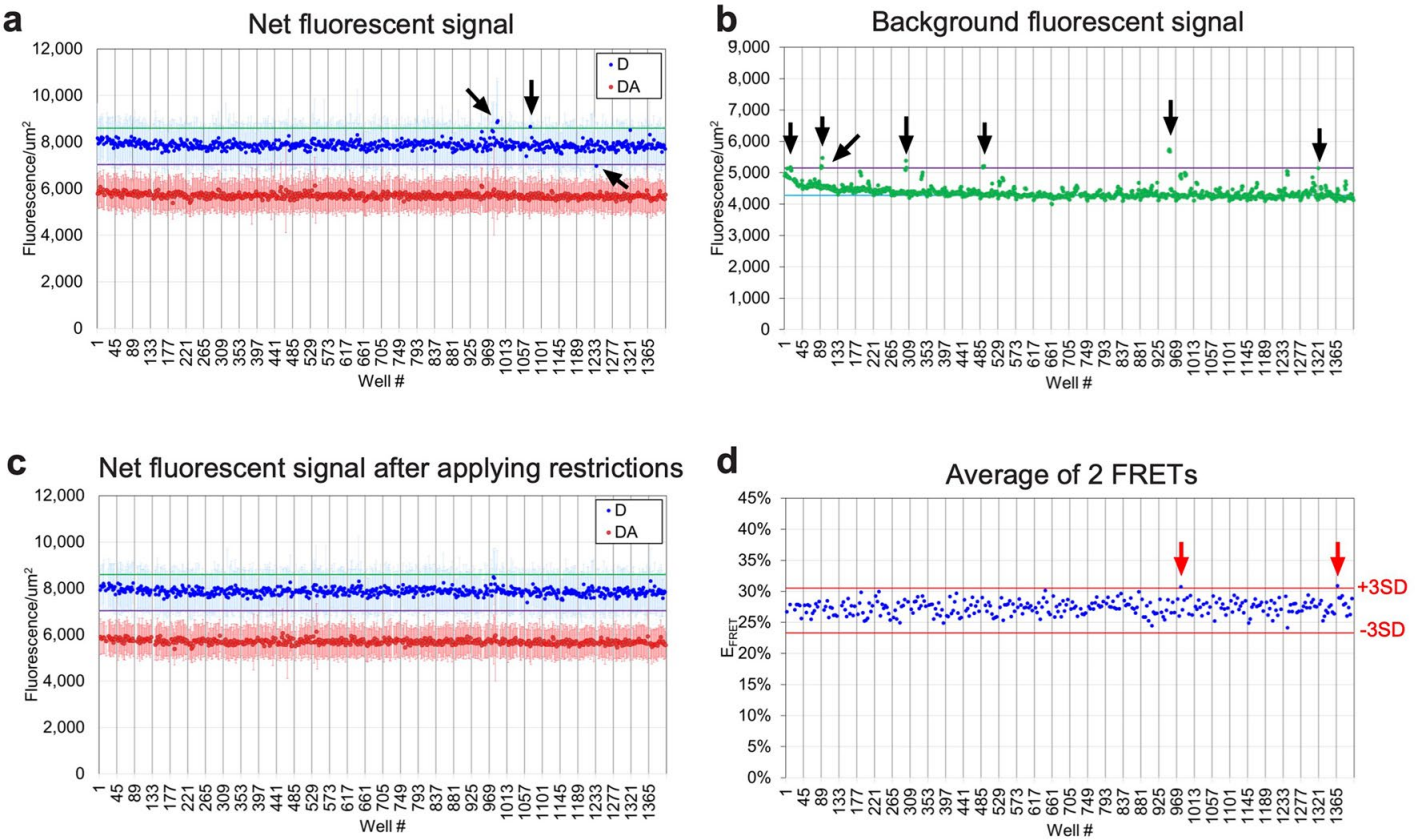
Representative plate analysis for 1,536-well HTS. (**a**) Graph showing bead fluorescence intensity of individual D (blue) or D + A (red) wells (mean ± SD, n =  ~ 64) in 1,536-well format. Arrows indicate sample where D fluorescence is above 1.1-fold (green line) or below 0.9-fold (magenta line) of mean of DMSO baseline control. (**b**) Graph showing background fluorescence intensity of each well. Black arrows indicate wells in which background signal was above 1.2-fold (magenta line) of mean background signal of DMSO baseline control (cyan line). (**c**) Graph showing fluorescence intensity of individual D (blue) or D + A (red) wells (mean ± SD, n =  ~ 64) after applying following two restrictions; donor fluorescence intensity was between 1.1-fold or 0.9-fold of DMSO control (shown in panel **a**); and background signal was below 1.2 fold of DMSO control (shown in panel **b**). (**d**) Individual blue dots represent E_FRET_ values calculated based on average of two D and D + A pairs from panel **c** for each compound. Red arrows indicate a hit compound showing averaged FRET values outside of ± 3 standard deviation (SD) of DMSO control (red lines in panels **d**).

**Figure 7:**
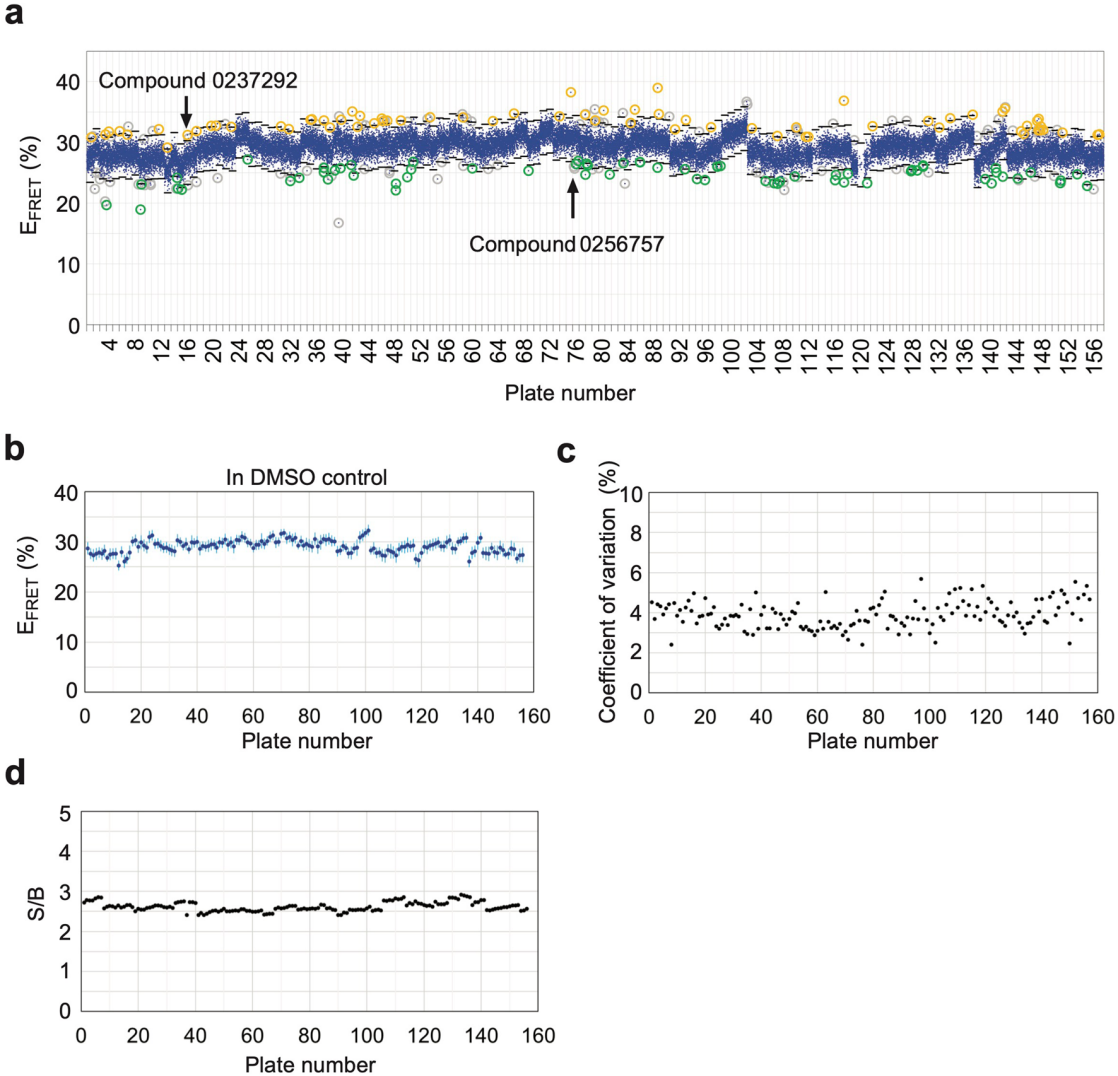
HTS results. (**a**) Raw FRET results for A455E NBD1 (acceptor and truncation at residues 487 and 654) screen using a 50,240 small molecule diversified library. Blue dots represent each averaged E_FRET_ value calculated based on two D and D + A pairs, and measured for compounds from 157 plates (1,536-well plate format, 600 beads/well, 320 compounds/plate). Black lines show ± 3SD for each plate. 133 primary hit compounds are indicated in orange circles (increase in FRET) or green circles (decrease in FRET) (0.26% hit ratio). Gray circles indicate averaged FRET values outside of ± 3SD but either or both single FRET values inside of ± 1.75SD. (**b**) Graph showing E_FRET_ values (mean ± SD, n = 32) of DMSO control for each plate. (**c**–**e**) Black dots represent coefficient of variation (SD/mean) of E_FRET_ values (**c**), or signal to background (mean D signal/mean background) (**d**) for each plate.

We tested a diverse 50,000 small-molecule library from ChemDiv, Inc. (https://www.chemdiv.com/) as a pilot HTS in 0.1% DMSO condition using batched and frozen RNCs expressing A455E NBD1 (acceptor probe and truncation sites at residues 487 and 654, respectively) in 1,536-well format. DMSO is well tolerated at the HTS condition (0.1% DMSO) (Supplementary Fig. S11). Compounds were tested in duplicate using paired D and D + A wells, thus allowing 320 compounds to be tested per plate (Supplementary Fig. S12). Figure 6 shows a representative plate analysis. To compensate for small molecule artifacts, compounds that increased or decreased baseline CFP fluorescence intensity and/or increased background signal intensity, were eliminated based on the following criteria as quality control (QC) tests: ≥  ± 10% change in fluorescence intensity of D beads or ≥ 20% change in background signal intensity (Fig. 6a–c). These criteria were chosen based on variations of bead signals and background signals. This resulted in approximately 10% of data being unusable. Note that no small molecules have been reported to directly interact with NBD1 cotranslational folding intermediates, therefore, this pilot HTS was performed without positive controls. Instead, DMSO baseline control data was used to identify hit compounds on the basis of averaged (N = 2) E_FRET_ measurements that showed >  ± 3SD relative to DMSO control (Fig. 6d). Figure 7a shows raw E_FRET_ values for ~ 50,000 compounds and 133 primary hit compounds (0.26% hit ratio) obtained from 157 1,536-well plates. 68 compounds out of the 133 hit compounds showed increase in E_FRET_ whereas other 65 compounds decreased E_FRET_. E_FRET_ values were remarkably consistent across plates measured on different days (Fig. 7b) as revealed for robust assay reproducibility (coefficient of variation (CV) = 3.9 ± 0.7% and signal to background ratio (S/B) = 2.62 ± 0.12) (Fig. 7c,d).

**Figure 8:**
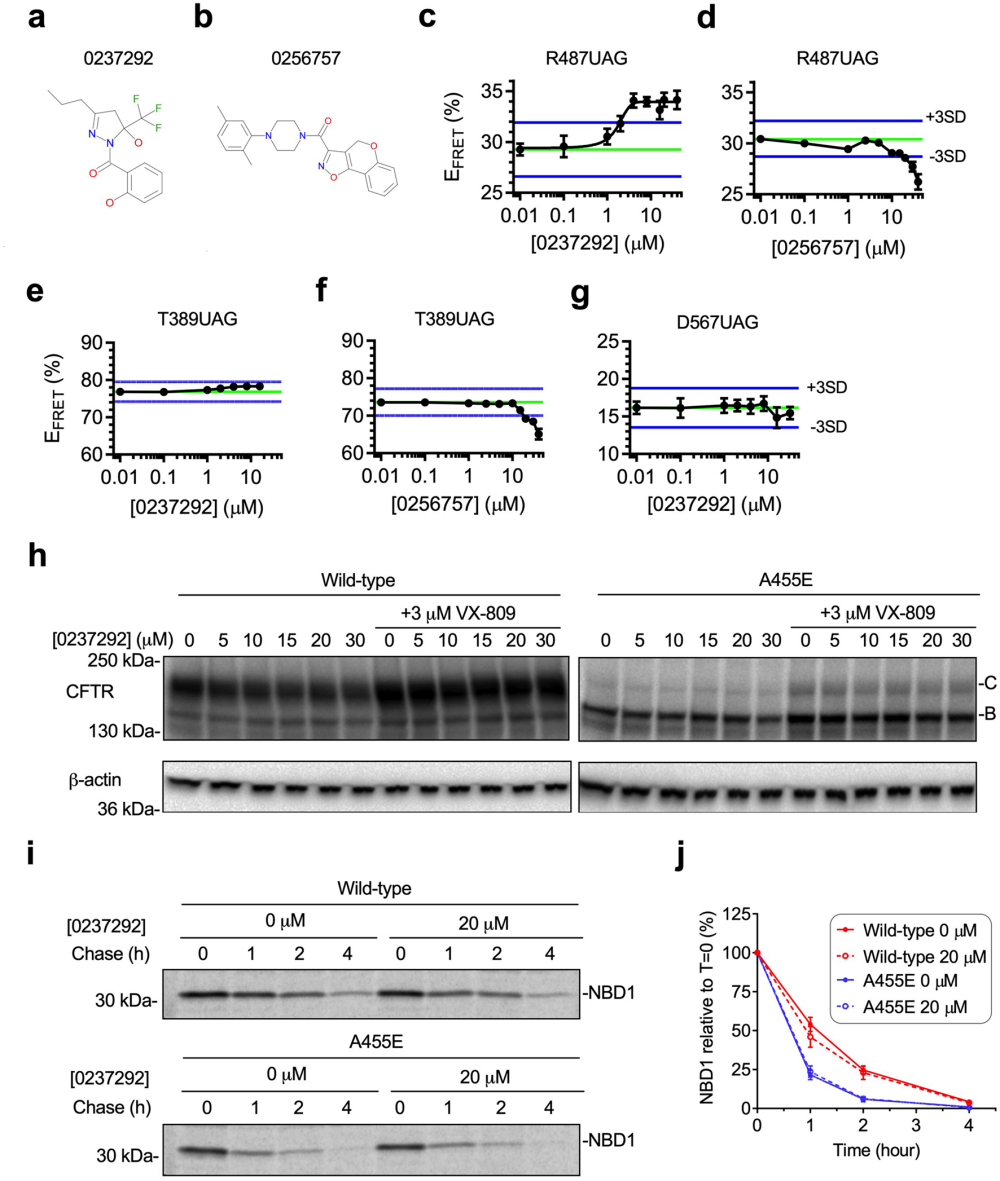
Hit confirmation. (**a, b**) Chemical structures of compound 023792 (**a**) or compound 0256757 (**b**). (**c**–**g**) Dose response results for compound 0237292 (**c**,**e**,**g**) or compound 0256757 (**d**,**f**) measured by solid-support FRET using A455E NBD1 RNCs with acceptor dye inserted at residue 487 (**c**,**d**), 389 (**e**,**f**), or 567 (**g**). Data are mean ± SEM, n ≥ 3 independent experiments. (**h**) Immunoblot of CFTR wild-type or A455E with 0–30 µM compound 0237292 with or without 3 µM VX-809 treatment expressed in human embryonic kidney (HEK) 293 cells showing no effect of compound 0237292 on both wild-type or A455E CFTR trafficking. Core glycosylated (band B) and mature CFTR (band C) are indicated. Uncropped blots in Supplementary Figure S15, S16. (**i**,**j**) Effect of compound 0,237,292 (20 µM) on stability of wild-type or A455E NBD1 in HEK 293 cells, determined by [^35^S]Met-labeled NBD1 immunoprecipitation. Uncropped gels in Supplementary Figure S17. Graph shows percentage of NBD1 recovered relative to T = 0 (mean ± SEM, n = 3 independent experiments).

All 133 primary hit compounds were subsequently tested in 6-point dose–response studies and two compounds were found to reproducibly altered E_FRET_ values (Fig. 8a–d). Compound 0237292 resulted in a dose dependent increase in E_FRET_ (5% at ≥ 4 µM with an EC_50_ of 1.7 µM) (Fig. 8c). In contrast, compound 0256757 decreased E_FRET_ values by 4% at 40 µM (Fig. 8d). To distinguish whether the observed changes in E_FRET_ reflected a direct effect on NBD1 folding, we used a previously described control NBD1 construct containing the acceptor probe at residue 389 (CFP fusion site). Recall that E_FRET_ for this construct is dependent only on the tether length. Therefore, compounds that specifically impact NBD1 folding should have no effect on E_FRET_. As shown in Fig. 8e, compound 0237292 did not substantially change E_FRET_ for the control construct, whereas compound 0256757 decreased E_FRET_ values to the same extent as were observed when the acceptor probe located at residue 487 (Fig. 8f). Thus, we suspect that compound 0256757 exerts its effect on fluorescent properties the donor and/or acceptor probes rather than NBD1 folding per se. Because the only difference between the control construct and screening constructs is the location of the probe with in the NBD1 domain (residues 389, and 487, respectively), we also conclude that compound 0237292 likely interacts with the nascent NBD1 polypeptide to produce the resulting increase in FRET. A455E mutation disrupts α/β-core formation during NBD1 cotranslational folding as described in our previous study using RNCs containing an acceptor probe at residue 567^24^ (Supplementary Fig. S13). To confirm whether compound 0237292 corrects folding disruption of α/β-core, we next tested compound 0237292 using the A455E NBD1 RNC construct containing an acceptor probe at residues 567 (truncation sites 674) as a secondary confirmation assay. However, compound 0237292 did not restore E_FRET_ (Fig. 8g). Taken together, compound 0237292 partially corrected A455E NBD1 folding intermediates, suggesting that additional restorations are needed to fully correct A455E folding defects.

Aside from the compound 0237292 identified in this pilot HTS, no small molecules have been shown to directly interact with NBD1 cotranslational folding intermediates. However, certain compounds, including several approved drug components, have been proposed to either interact with full-length CFTR or to influence cotranslational folding outcome through unknown mechanisms. We therefore examined 5-bromoindole-3-acetic acid (BIA), a reported NBD1 stabilizer^54^, and other corrector molecules including VX-809, VX-661, and VX-445 in our cotranslational folding assay. Supplementary Figure S14 showed that none of these compounds restored E_FRET_ for RNCs of A455E NBD1, although BIA decreased CFP fluorescence intensity at the higher concentrations (≥ 4 mM).

We next tested whether compound 0237292 would correct folding of A455E NBD1 or full-length CFTR. As shown in Fig. 8h, compound 0237292 did not influence either wild-type or A455E CFTR trafficking in HEK293 cells as indicated by lack of Golgi-processed, Band C protein. VX-809 (lumacaftor), which partially restores CFTR folding, was added if compound 0237292 showed any synergistic effects with VX-809. However, addition of 3 µM VX-809 to compound 0237292 treatment failed to show any additional effects. Pulse-chase experiments also revealed that 0237292 had no significant effect on stability of wild-type or A455E for the isolated NBD1 domain (two-tailed student’s t-test for protein half-life, p = 0.32 or 0.64, respectively) (Fig. 8i,j).

## Discussion

In this study we report a highly sensitive solid-support assay system well suited for high throughput screening that is capable of detecting subtle conformational changes in ribosome bound nascent proteins. Quantitative fluorescence imaging of immobilized RNCs generated by cell free in vitro translation allowed detection of approximately 0.4 attomole RNCs/bead with high degree of reproducibility in 1536 well format (CV = 3.9 ± 0.7%). RNCs isolated from cell free in vitro translation reactions are stable at room temperature and amenable to cryopreservation, enabling scaling efforts needed for screening consistency. A pilot HTS of approximately 50,000 small molecules in 1,536-well plate format demonstrated suitability for screening small molecules that interact with, and alter nascent protein structure. These results provide a unique HTS method to capture folding intermediates of both wild-type and genetic mutants that are normally present only transiently during synthesis. In addition, it is also suited for full length or truncated proteins regardless of their ribosome attached or released state.

Our pilot HTS identified a single validated hit (#0,237,292) that restored E_FRET_ for RNCs of A455E NBD1 containing an acceptor probe at residue 487 to the same extent as previously described suppressor mutations (Fig. 8c, e). Unfortunately, activity of 0,237,292 did not translate into improved CFTR trafficking or NBD1 stability (Fig. 8h-j). This is perhaps not surprising because compound 0,237,292 partially corrected A455E NBD1 folding defects (Fig. 8g) and the molecule itself could affect numerous other aspects of CFTR folding as has been proposed for compounds that increase NBD1 thermal stability^32,54,55^. Although disappointing, the likelihood of identifying a compound that would act with this specific mechanism of action from a relatively small 50,000 compound library is quite low. Libraries covering a much larger chemical space would likely be needed to identify small molecules that act via such a mechanism.

We acknowledge that one of the most technically challenging aspects of this system involves generation of biochemically uniform cohorts of RNCs with defined fluorophore stoichiometry. Fluorophore incorporation is also restricted to peptide regions that will tolerate modification by a donor and acceptor probe. However, the growing number of studies using FRET to monitor conformational changes associated with folding, function, enzyme activation, posttranslational modification, and oligomerization, support a broader applicability for this approach^56–61^.

It is also important to note that techniques reported here could potentially be further optimized to improve in vitro translation yield, solid support capture efficiency, and uniformity in substrate binding. Of the 12 binding strata tested, we found that Ni-IDA 17 µm beads gave the best signal, binding efficiency, and fluorescence reproducibility. Bead-binding conditions shown here for 17 µm beads yielded quite acceptable signal-to-background ratios and achieved surface densities of 250–500 RNCs/µm^2^. This represents 13–27% theoretical geometric saturation based on ribosome cross-sectional area and an effective increase in RNC concentration approximately 100-fold greater than solution studies (~ 2 nM in solution vs. 0.16–0.31 µM bound). RNC immobilization therefore enabled us to achieve sub-attomolar detection sensitivity. Typical translation reactions of the His_10_-CFP-NBD1 A455E (acceptor 487 and truncation 654) construct used in this study (6 ml cell free in vitro translation reactions) yield sufficient material (42 picomol of purified RNCs) to generate approximately 26 million beads (13 million each of D and D + A beads), which when plated at 600 beads/well in 1,536-well plate, provides sufficient material for 21,500 single FRET measurements. Although this method relies on image-based readout like high-content screening (HCS) systems, with an image time of 0.5 s per well (1-image/well) in 1,536-well plate format using the GE IN Cell Analyzer 2200, approximately 1 million small molecule compounds could theoretically be screened in 3 months (36 assay plates a day), which meets the HTS standard (> 100,000 compounds screen)^62^. We would also note that in our experience, translation yields vary for different protein constructs, and are dependent upon the length of transcript and location and readthrough efficiency of the stop codon (~ 30% in this study). However, our results reflect reasonable estimates that were readily obtained and which could be further optimized or scaled if needed for industrial screening programs.

Important practical considerations include: (i) the time required from RNA transcription to protein synthesis and RNC purification is approximately 4 h; (ii) no large-scale protein purification is required; (iii) partial length and even misfolded proteins are stably retained, while bound to the ribosome, in a non-aggregated, folding-competent state for prolonged periods of time ^1,2,44^. Immobilized RNCs are therefore well suited for substrates that cannot be efficiently expressed in cells and/or are not amenable to traditional biochemical analyses for other reasons. Importantly, there are currently no existing HTS assays to evaluate cotranslational folding, which represents a potential novel approach for developing new treatment strategies^24^. Thus, the HTS assay reported here could be useful in studying additional CFTR mutations or adapting for use in other protein folding disorders or drug discovery efforts.

## Methods

### Plasmids

eCFP-NBD1 fusion constructs containing UAG stop codons at CFTR residues Thr389, Arg450, or Arg487 were generated in the pSP64 plasmid vector (Promega) as described previously^1^. A His_10_ tag (5’-catcaccatcaccatcaccatcaccatcac) followed by flexible 21 amino acid residues linker (GGS)_7_ was fused the N-terminus of CFP by PCR overlap extension as described elsewhere^1^. A455E mutation was generated in a similar technique^24^. All cloned PCR fragments were verified by DNA sequencing.

### In vitro transcription and translation

Noncapped RNA transcripts were synthesized from PCR-amplified DNA templates (15 ng/μl) in 80 mM HEPES–NaOH (pH 7.5); 16 mM MgCl_2_; 2 mM spermidine; 3 mM each ATP, CTP, UTP, and GTP; 10 mM DTT; 0.2 U/μl RNase inhibitor; and 5 μg/ml SP6 RNA polymerase at 40 °C for 2 h as described elsewhere^63^. RNA was precipitated with 3 M LiCl and 20 mM EDTA at -20 °C for 1 h and centrifuged at 16,000 × *g* at 4 °C. The RNA pellet was washed three times with 70% (v/v) ethanol and then resuspended in RNase free H_2_O and stored at − 80 °C.

Four parallel in vitro translation reactions were performed as described previously^1,2^. Briefly translation was carried out for 72 min at 24 °C in reactions containing 60 ng/μl purified RNA, 40% rabbit reticulocyte lysate (RRL) prepared as described previously^63^, 20 mM HEPES–KOH (pH 7.6), 100 mM KOAc, 1.6–2.0 mM Mg(OAc)_2_, 50 μM each of 20 amino acids, 1 mM ATP, 1 mM GTP, 15 mM creatine phosphate, 2 mM DTT, 0.15 mM spermidine, 40 ng/μl bovine tRNA, 40 ng/μl creatine kinase, 0.12 U/μl RNase inhibitor, and either 1 μM [^14^C]Lys-tRNA^amb^ or εN-7-nitrobenz-2-oxa-1,3-diazol (εNBD)-[^14^C]Lys-tRNA^amb^ prepared as described previously^1^. Matched translation samples were prepared as following: CFP donor only (D)—translated in the presence of [^14^C]Lys-tRNA^amb^, Donor + Acceptor (D + A)—translated in the presence of εNBD-[^14^C]Lys-tRNA^amb^, and blank-D and blank-D + A (BD and BDA)—two “blank” (control) reactions in the presence of either [^14^C]Lys-tRNA^amb^ or εNBD-[^14^C]Lys-tRNA^amb^ were prepared using non-fluorescence eCFP expressing transcripts lacking a UAG codon as described previously^2^. Blank samples were used to quantify the concentration of ribosome nascent chain complexes (RNCs) to use for bead binding reactions, and to obtain ^14^C-corrected fluorescence intensity in solution-based FRET measurement. An RNA aptamer that inhibits translation termination factors (eRF1/eRF3) was added (0.2–2 μM) to translation reaction to improve read-through and achieve similar readthrough efficiencies for D and D + A translation reactions^2,64^.

### RNCs preparation

RNCs were isolated from translation reactions at 4 °C by size exclusion column chromatography (Sepharose CL-6B) equilibrated in buffer containing 40 mM HEPES–KOH (pH 7.6), 100 mM KOAc, and 10 mM MgCl_2_ (column buffer), flash frozen in liquid nitrogen, and store at − 80 °C. RNC concentration was determined by ^14^C scintillation counting using the following equation:

2 $$\left[ {{\text{RNC}}} \right] = \left( {{\text{cpm}}_{{\text{S}}} - {\text{cpm}}_{{\text{B}}} } \right)/\left( {{\text{CE }} \times {\text{ SA}} \times {\text{vol}}} \right)$$

where [RNC] is the concentration of RNCs (in nM), cpm_S_ and cpm_B_ are ^14^C counts/min of sample and blank sample (D and BD, or D + A and BDA pair), respectively. CE is counting efficiency (estimated at 95% for ^14^C), SA is the specific activity of [^14^C]Lys in dpm/pmol, and vol is the volume of sample in ml.

### Solution based fluorescence measurements

Fluorescence measurement and calculation of FRET efficiency were performed as described previously^1,2^. CFP fluorescence emission spectra (λex = 430 nm, λem = 450–600 nm, 1 nm intervals) obtained from purified RNCs were measured at 23 °C using a Fluorolog 3–22 fluorometer (HORIBA Jobin Yvon, Edison, NJ). 5 highest peak emission intensities (~ 475 nm) were averaged and used for subsequent calculation. Ribosome release was performed by adding column buffer with final concentrations of 200 μg/ml RNase A and 3 mM ATP. At the end of measurement, the concentration of polypeptide in D and D + A samples was determined by ^14^C scintillation counting using the Eq. (2). FRET efficiency was then calculated by the acceptor-dependent decrease in CFP fluorescence intensity from the Eq. (1) as described in “Results” section.

### Bead binding and assay plate preparation

RNC binding to beads was carried out in 400 μl binding buffer (column buffer plus 0.5 mM DTT and 20 mM Imidazole–HCl pH 7.2) in a Protein LoBind round-bottom polypropylene tube 2.0 mL (Eppendorf) using a Fisher Scientific 346 Hematology/Chemistry Mixer at room temperature. Typical conditions involved 2 nM RNCs, 6 h incubation time length, and 2 × 10^5^ of *High Density Nickel 4 Highly Cross-linked Superfine 17 µm* (Agarose Bead Technologies, 17 µm beads), 5 × 10^4^ of *HiTrap Chelating HP* (GE Healthcare, 34 µm beads) charged with Nickel as described elsewhere^65^, or 6 × 10^3^ of *Ni–NTA agarose* (Qiagen, 100 µm beads) unless otherwise stated. For high-throughput screening (HTS), bead binding was carried out with 2 nM RNCs, and 1 × 10^6^ of 17 µm beads in 800 μl binding buffer over-night (16 h) at room temperature. Beads were then transferred to V-bottom shaped 1.5 mL Eppendorf tube and washed × 3 with 1 ml of binding buffer followed by centrifugation at 5,000 × *g* at room temperature for 1 min. D and D + A beads were mixed in 50 mL conical tubes with 6.7 ml of column buffer plus 0.5 mM DTT (150 beads/µl at final concentration). 1,536 well (or 96/384 well for earlier experiments) glass bottom plates (Greiner Bio-One, Cat# 783892 or Cellvis, Cat# P96/384-1.5H-N) were used as assay plates. 600 beads, 500 beads, or 1,000 beads were dispensed into each well of 1,536 well, 384 well, or 96 well plates, respectively, by Multidrop Combi Reagent Dispenser (Thermo Scientific) and allowed to settle to well bottom by gravity for 30 min. Final assay volumes were adjusted to 7.5 µl, 50 µl, or 200 µl in each well by dispensing column buffer plus 0.5 mM DTT into 1,536 well, 384 well, or 96 well plates, respectively, prior to dispensing bead solution. Where indicated, nascent chains were released from ribosomes by addition of 200 μg/ml RNase A and 3 mM ATP at final concentrations. RNC surface density was calculated using number of total protein molecules bound (derived from ^14^C counting using the Eq. (2) and total surface area of beads as described in “Results” section. Bead binding for free nascent chain was performed by preincubating RNCs incubated with RNase A (0.2 mg/ml) for 15 min at room temperature prior to incubation with beads.

### HTS

50,240 small molecule compounds from a diversified library (ChemDiv, inc.) were tested in this study. 7.5 nl of 10 mM compounds in 100% DMSO were dispensed into each well of 1,536 well plates (Greiner Bio-One, Cat# 783892) by Echo 550 Liquid Handler (Beckman Coulter). 3.5 µl of column buffer plus 0.5 mM DTT was then mixed with the compounds each well followed by centrifugation at 500 × *g* at room temperature for 1 min. 4 µl of bead solution (150 beads/µl) was dispensed into each well of assay plates by Multidrop Combi Reagent Dispenser (Thermo Scientific). Each assay plate includes the vehicle control wells (0.1% DMSO) as showing in Supplementary Figure S12. 6-point dose–response assays (5–40 µM compound, 0.4% DMSO) were performed for primary hit compounds in the same procedures as the HTS.

### Imaging and analysis

Beads were imaged in column buffer plus 0.5 mM DTT at 25 °C for all experiments. Three microscopes were tested in developing this protocol as described below; In Cell Analyzer 2200 software version 4 or 6 (GE Healthcare) [excitation wavelength, 438 nm (426–450 nm), emission wavelength, 475 nm (463–487 nm), 20 × 0.75NA objective, 0.325 × 0.325 nm^2^/pixel, 1 or 0.5 s exposure, 1, 4, or 9 images per well]; Nikon Ti-E eclipse microscope with Lumencor Sola Light Engine and Andor Zyla Camera [excitation wavelength, 436 nm (426–446 nm), emission wavelength, 480 nm (460–500 nm), 20 × 0.75NA objective, 1 s exposure]; and Olympus IX71 high resolution widefield inverted microscope and features an LED transmitted light source for differential interference contrast [excitation wavelength, 430 nm (418–442 nm), emission wavelength, 470 nm (458–482 nm), 3 s exposure]. While all the three microscopes can be used to detect fluorescence signal of beads from images, the GE In Cell System was superior for HTS. For GE In Cell Analyzer, flat-field correction was applied to minimize well-to-well and in-well variations. Note that flat-field corrections between instrument software version 4 and 6 are not identical due to different algorithms of software, and provide substantial differences in the signal intensity (fluorescence units). This did not have effect FRET measurements as showing in Supplementary Figure S6. The central focal plane of the image was set to the distance of bead radius from the bottom of the well. HTS was carried out in 1,536 well plate format (1 image/well, 0.5 s exposure). Earlier experiments were carried out in 96 or 384 well plates (9 images/well, 1.0 s exposure). Valid bead images were selected using In Cell Developer Toolbox software v1.9 (GE Healthcare), with image segmentation method as described below. Unfocused beads and artifact objects were eliminated by applying restrictions of diameter (16–23 μm for 17 µm beads), circularity (> 0.93), and threshold pixel intensity. Mean fluorescent intensity of selected beads was quantitated per unit area, and data was analyzed using excel and/or prism software. FRET efficiency was calculated by the Eq. (1) as above. For earlier experiments, the Z’ factor^53^ was calculated from the following equation:

3 $${\text{Z}}^{\prime} \, = {1} - {3}\times\left( {{\text{SD}}_{{\text{P}}} + {\text{ SD}}_{{\text{N}}} } \right)/\left( {{\text{Mean}}_{{\text{P}}} - {\text{ Mean}}_{{\text{N}}} } \right)$$

where SD_P_ and SD_N_ are standard deviation of positive and negative controls, respectively. Mean_P_ and Mean_N_ are mean of positive and negative controls, respectively. For practical purposes, positive control is ΔRI and negative control is wild-type.

### Immunoblotting

Human embryonic kidney (HEK) 293 cells (ATCC, Cat# CRL-1573) were grown at 37 °C under 5% CO_2_ in Dulbecco's modified Eagle's medium (Invitrogen) supplemented with 10% fetal calf serum (Thermo Scientific) and penicillin/streptomycin (Invitrogen). 5 × 10^5^ cells were seeded in six-well plates and transfected one day later with equal amounts (2.5 μg) of pcDNA3-CFTR vector containing wild-type or A455E as indicated. Cells were treated with compounds (final 1% DMSO) 24 h after transfection and harvested 24 h after compound treatment by lysis for 20 min in 600 μl of ice-cold RIPA buffer (20 mM HEPES- NaOH/pH 7.5, 150 mM NaCl, 1 mM EDTA, 1% Triton X-100, 0.1% SDS, 0.5% sodium deoxycholate) containing cOmplete Protease Inhibitor Cocktail (Roche Applied Science). Cell lysate was separated by SDS–PAGE, transferred to PVDF membrane (Millipore), and immunoblotted using the following primary antibodies: (1) mouse anti-CFTR antibody M3A7 (Millipore, Cat# 05-593, Lot# 2652963, 1:2,000 dilution), and (2) rabbit anti-β-actin (Santa Cruz Biotech., Cat# sc-47778, Lot# B1914, 1:2,000 dilution) and secondary antibodies: (3) goat anti-mouse IgG (H + L)-HRP conjugate (Bio-Rad, Cat# 1706516, 1:5,000 dilution), or (4) goat anti-rabbit IgG-HRP (Santa Cruz Biotech., Cat# sc-2030, 1:5,000 dilution). Blots were imaged using the ChemiDoc XRS + System (Bio-Rad) and analyzed using accompanying image analysis software.

### [^35^S]-methionine pulse-chase labeling

HEK 293 cells were grown at 37 °C under 5% CO_2_ in Dulbecco's modified Eagle's medium (Invitrogen) supplemented with 10% fetal calf serum (Thermo Scientific) and penicillin/streptomycin (Invitrogen). 5 × 10^5^ cells were seeded in six-well plates and transfected one day later with equal amounts (2.5 μg) of pcDNA3-NBD1 vector containing wild-type or A455E as indicated. Cells were treated with or without compound treatment (final 1% DMSO) 24 h after transfection, incubated for 1 h in cysteine and methionine-free medium with the compound 1 h after compound treatment, pulsed labeled with 30 μCi Trans^35^S-label (MP Biomedicals)/well with the compound for 30 min, chased with the regular media and the compound for 0–4 h, and harvested directly at the indicated times by lysis for 20 min in 600 μl of ice-cold RIPA buffer containing complete protease inhibitor mixture. Cell lysate was incubated with anti-CFTR 3G11 rat monoclonal antibody (Cystic Fibrosis Folding Consortium) overnight at 4 °C, and then for 2 h at 4 °C after addition of Affi-gel Protein G (Bio-Rad). Protein G beads were washed five times with RIPA buffer and mixed with SDS-PAGE sample buffer, followed by separation on SDS-PAGE. The radio-labeled bands were imaged and analyzed with the Personal FX phosphor imager and Quantity One software (Bio-Rad).

## Supplementary Information

Supplementary Information.

## Data Availability

All data in this study are available from the corresponding author upon reasonable request.
