# Supplementary material for: A small molecule high throughput screening platform to profile conformational properties of nascent, ribosome-bound proteins
Source: Sci Rep. 2022 Feb 15;12:2509. doi: 10.1038/s41598-022-06456-5 (PMC8847357; doi:10.1038/s41598-022-06456-5)
Supplement: Supplementary file 1 — Supplementary Information. [file 41598_2022_6456_MOESM1_ESM.pdf]

A high throughput screening platform to interrogate cotranslational protein folding intermediates using immobilized ribosome nascent chain complexes

Hideki Shishido[1, 3], Jae Seok Yoon[1], and William R. Skach[2*]

[1]CFFT Lab, Cystic Fibrosis Foundation, 44 Hartwell Ave. Lexington, MA 02421, USA.
[2]Cystic Fibrosis Foundation, 4550 Montgomery Ave., Suite 1100N, Bethesda, MD 20814, USA.
[3]Current affiliation: Generate Biomedicines, Inc., 26 Landsdowne St. Cambridge, MA 02139, USA.

*Correspondence and requests for materials should be addressed to W.R.S. (E-mail: wskach@cff.org)

This pdf file includes:

Supplementary Figures S1 to S17

Supplementary Table S1

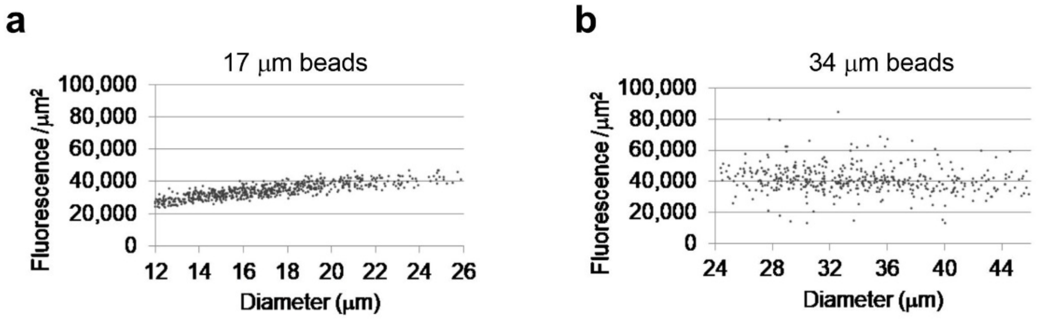

Supplementary Figure S1. Variation of fluorescence signal of 17 μm and 34 μm beads. Scatter plot showing fluorescent intensity of individual beads containing His_{10}-CFP-NBD1 (acceptor 450 and truncation 550) plotted against bead diameter using 17 μm (**a**) and 34 μm (**b**) beads.

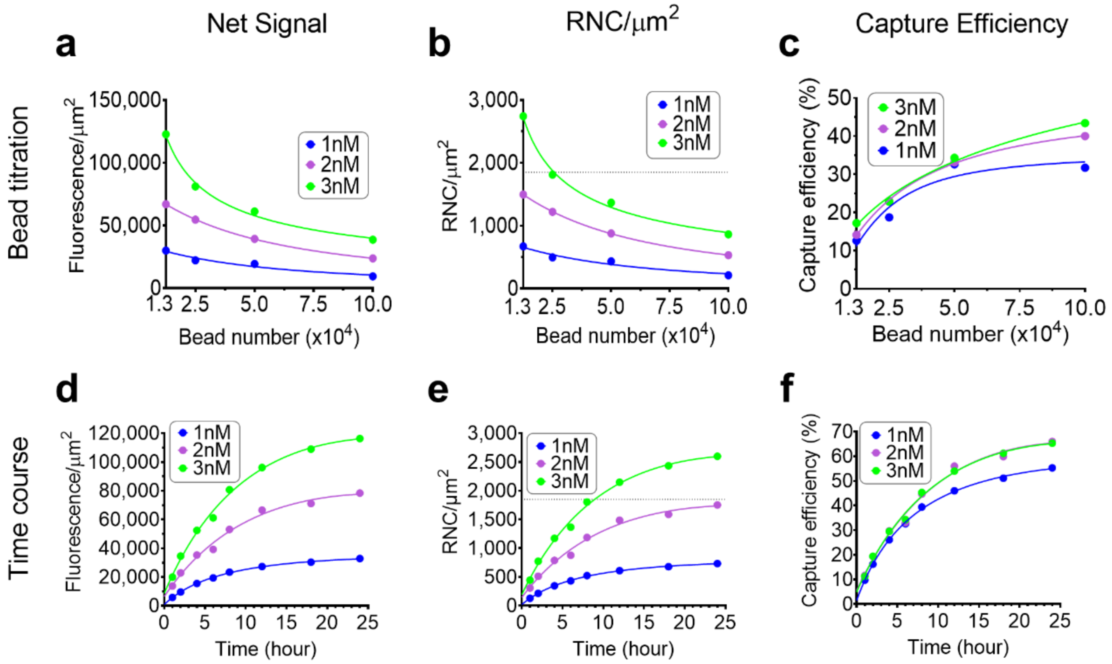

Supplementary Figure S2. RNC capture is dependent on RNC concentration, binding time, and bead number using 34 µm beads. (a-c) Effect of bead number on RNC binding. 1, 2, or 3 nM RNCs (His_{10}-CFP-NBD1, donor only) were incubated with 1.25-10 x 10^4 of 34 µm beads for 6 hours. (**d-f**) Effect of incubation time on RNC binding. 1, 2, or 3 nM RNCs (His_{10}-CFP-NBD1 donor only) incubated with 5 x 10^4 of 34 µm beads. Binding density was calculated using number of protein molecules bound per total calculated surface area of beads added. Dotted line in panel **b** and **e** indicates theoretical RNC saturation density (~1,850 RNCs/µm^2 as described in **Fig. 2b**). Capture efficiency was calculated by RNCs bound/RNCs in binding reaction.

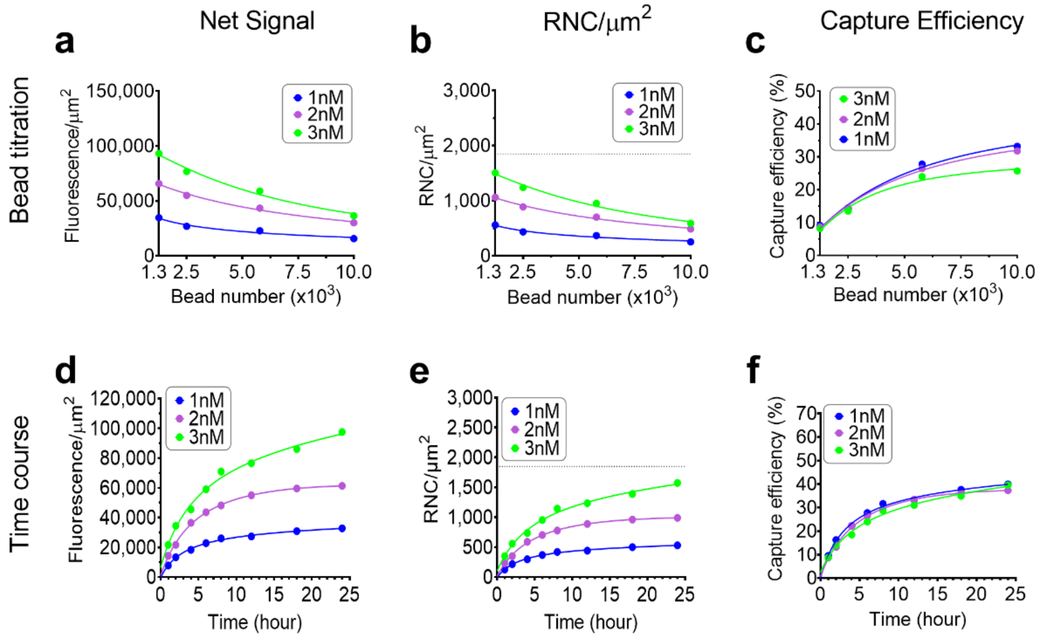

Supplementary Figure S3. RNC capture is dependent on RNC concentration, binding time, and bead number using 100 μm beads. (a-c) Effect of bead number on RNC binding. 1, 2, or 3 nM RNCs (His_{10}-CFP-NBD1, donor only) were incubated with 1.25-10 x 10^3 of 100 μm beads for 6 hours. (**d-f**) Effect of incubation time on RNC binding. Binding density was calculated using number of protein molecules bound per total calculated surface area of beads added. Dotted line in panel **b** and **e** indicates theoretical RNC saturation density (~1,850 RNCs/μm^2 described in **Fig. 2b**). Capture efficiency was calculated by RNCs bound/RNCs in binding reaction.

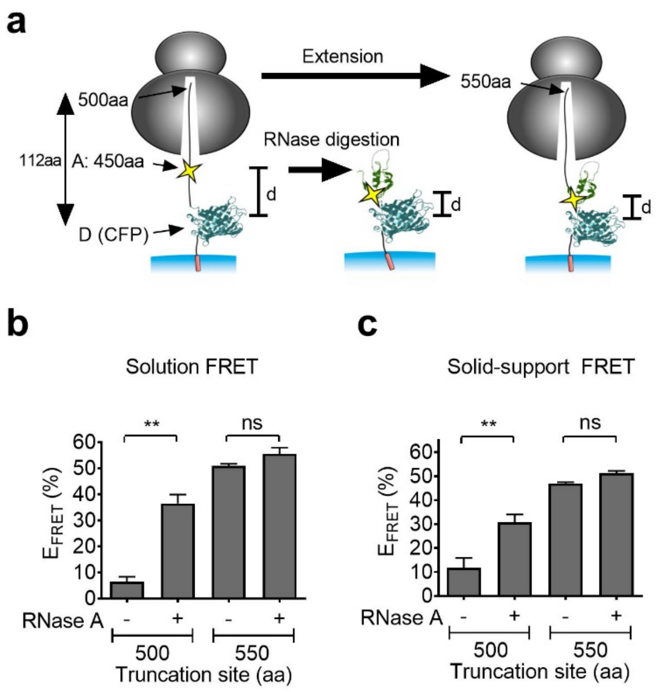

Supplementary Figure S4. RNC immobilization dose not statistically impact nascent NBD1 folding intermediates. (**a**) Cartoon showing His_{10}-CFP-NBD1 fusion proteins (acceptor 450 and truncation 500) isolated on beads in the unfolded state before and after ribosome release, and His_{10}-CFP-NBD1 fusion proteins (acceptor 450 and truncation 550) isolated in the folded state ribosome bound. (**b** & **c**) Solution FRET results (**b**), or solid-support FRET of 17μm beads (**c**) using constructs shown in panel **a** (n ≥3 independent experiments ± SEM). Two-tailed paired student's t-test, **p < 0.01, n.s. > 0.05. Results show FRET increase following ribosome release (500 truncation), whereas at truncation 550, N-terminal subdomain has already folded on the ribosome.

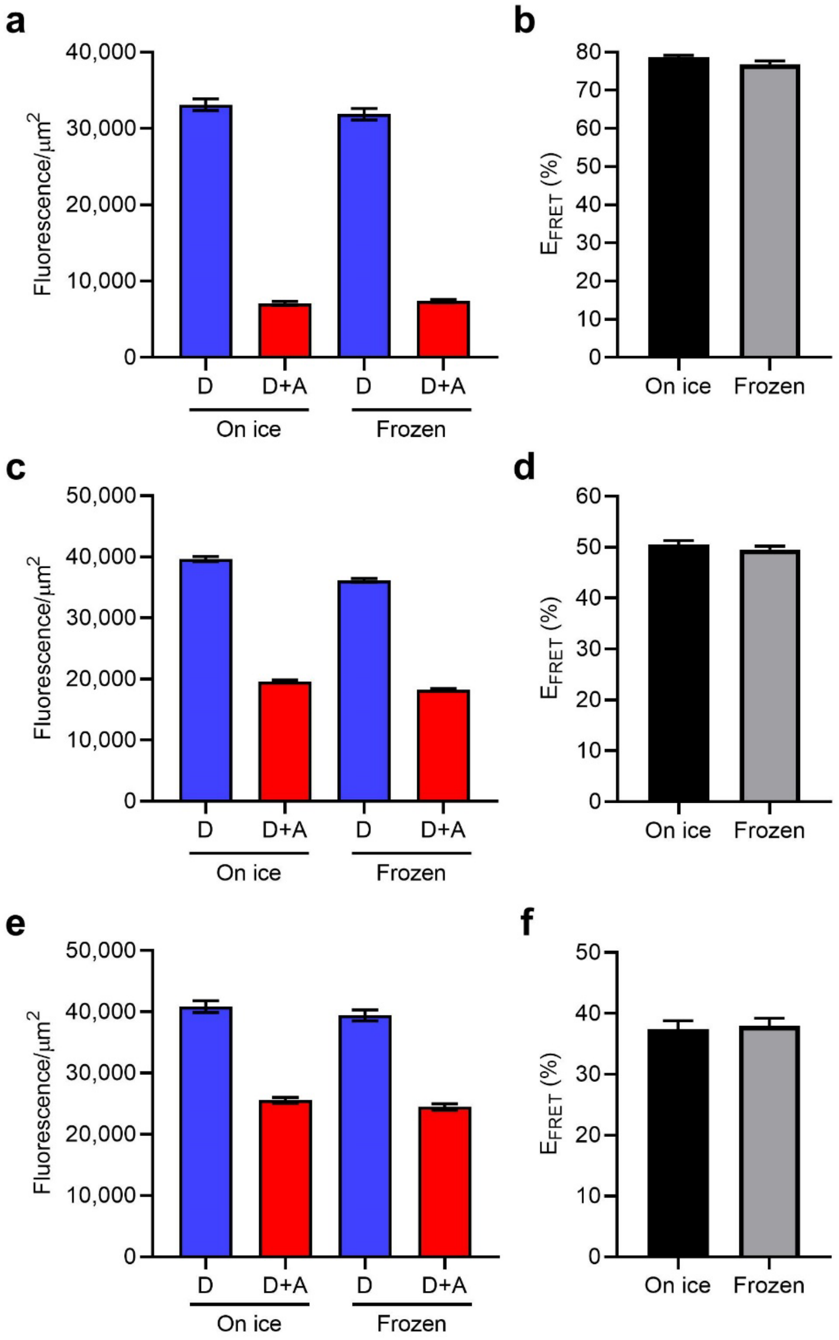

Supplementary Figure S5. Freeze-thaw effects on nascent chain folding. Fluorescence intensities of beads containing bound D or D+A RNCs (mean ± SD) (**a, c, e**) and E_{FRET} (n ≥3 ± SEM) (**b, d, f**) measured for fresh or frozen RNCs of His_{10}-CFP-NBD1 with acceptor probe and truncation at residues 389 and 500 (**a**, **b**), 450 and 550 (**c**, **d**), or 487 and 654 (**e**, **f**), respectively.

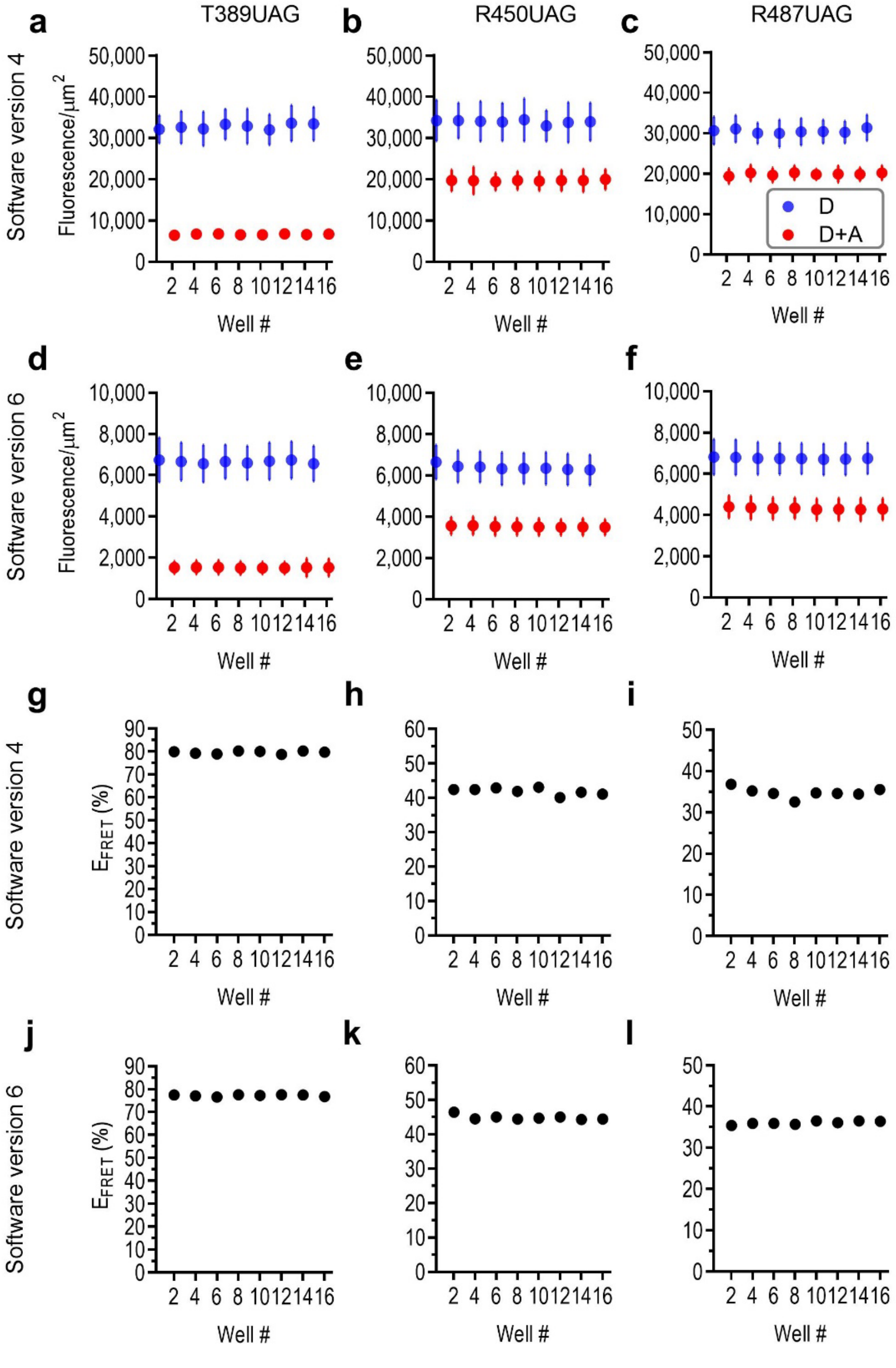

Supplementary Figure S6. **Two software versions used in this study yield different fluorescence intensities but similar FRET values.** (**a-f**) Individual dots show mean ± SD of D (blue) and D+A (red) fluorescence intensities in matched paired wells for each RNC constructs indicated at top measured by In Cell Analyzer 2200 software version 4 (**a-c**) or 6 (**d-f**). (**g-l**) Individual data show FRET values calculated based on each D and D+A pair in panel **a-f**, respectively.

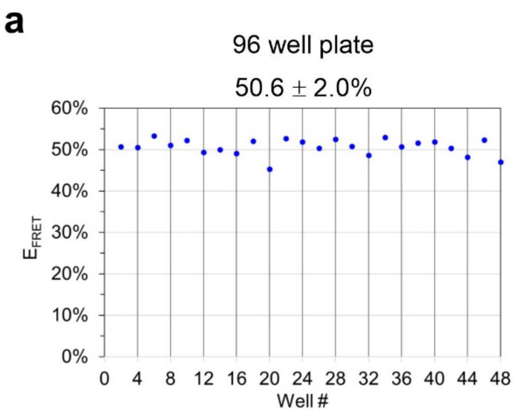

Supplementary Figure S7. Solid-support FRET in 96 and 384 well plate formats. Individual data show FRET values (mean ± SD) using 17μm beads in a 96 well plate (1,000 beads/well) (**a**) or a 384 well plate (500 beads/well) (**b**). 48 wells were used for each plate. 4 images were obtained per well. Construct was His_{10}-CFP-NBD1 (acceptor 450 and truncation 550).

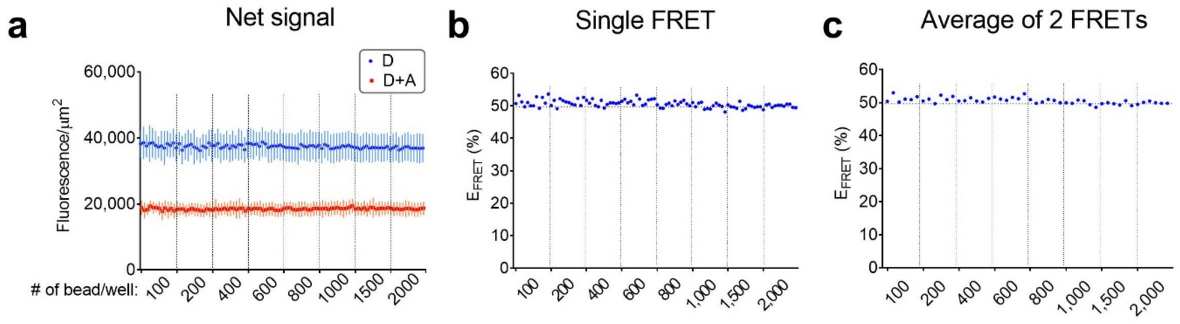

d

# of beads/well	100	200	400	600	800	1,000	1,500	2,000
Single FRET	51.2 ± 1.5%	51.1 ± 1.1%	50.8 ± 0.8%	51.5 ± 0.9%	50.4 ± 0.8%	49.9 ± 1.0%	49.7 ± 0.7%	49.9 ± 0.5%
Average of 2 FRETs	51.2 ± 1.0%	51.1 ± 0.9%	50.8 ± 0.5%	51.5 ± 0.7%	50.4 ± 0.5%	49.9 ± 0.8%	49.7 ± 0.5%	49.9 ± 0.3%

Supplementary Figure S8. Effect of bead number on reproducibility in 384-well plate format. (**a**) Titration of bead number in 384 well plate for 17µm beads (100-2,000). Results show mean ± SD of D and D+A fluorescence intensities in matched paired wells. 12 wells were used for each bead number well indicated at bottom. 9 images were obtained per well (=37% of total well area). Construct was His_{10}-CFP-NBD1 (acceptor 450 and truncation 550). (**b**) E_{FRET} values for each D and D+A pair. (**c**) E_{FRET} values calculated based on average of two D and D+A pairs. (**d**) Summary of E_{FRET} results (mean ± SD) for single and average FRET measurements for each ranged dispensed bead number.

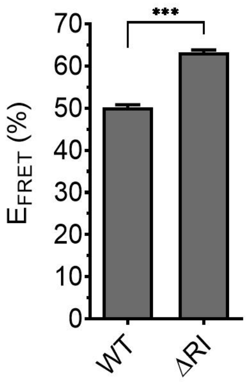

Supplementary Figure S9. Deletion of regulatory region (ΔRI) increased FRET efficiency of wild-type RNC. Mean E_{FRET} values obtained for wild-type or ΔRI NBD1 RNCs (acceptor 450 and truncation 550) in 384 well plate format (400 beads/well).

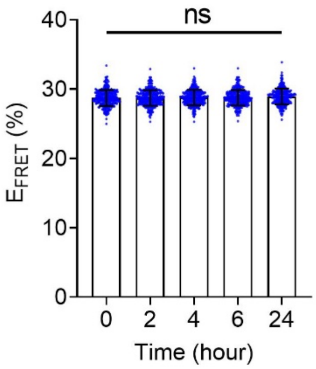

Supplementary Figure S10. Stability of RNCs for solid-support FRET. Solid-support FRET for the A455E NBD1 RNCs (acceptor 487 and truncation 654) measured multiple times using the same 1,536-well plate after incubation at 24°C for the indicated times (mean ± SD). Two-tailed student's t-test, ns > 0.05.

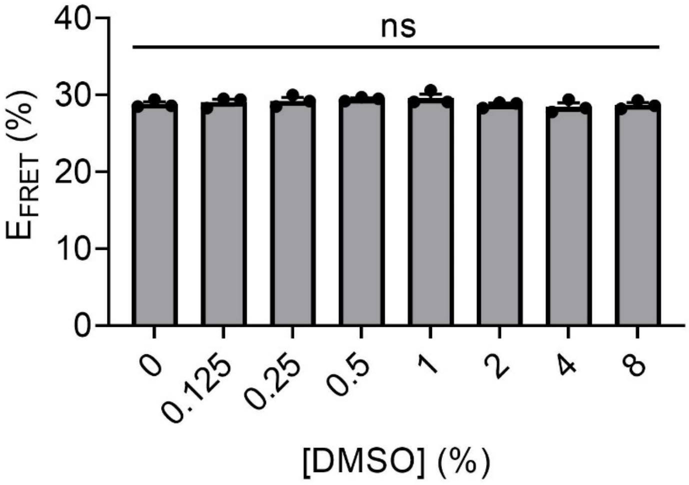

Supplementary Figure S11. DMSO tolerance of solid-support FRET. Solid-support FRET for the A455E NBD1 RNCs (acceptor 487 and truncation 654) measured in 0-8% DMSO (mean ± SEM, n=3 independent experiments). Two-tailed student's t-test, ns > 0.05.

	1	2	3	4	5	6	7	8	9	10	11	12	13	14	15	16	17	18	19	20	21	22	23	24	25	26	27	28	29	30	31	32	33	34	35	36	37	38	39	40	41	42	43	44	45	46	47	48	
A						#1				#2				#3				#4				#5				#6				#7				#8				#9				#10							A
B						#11				#12				#13				#14				#15				#16				#17				#18				#19				#20							B
C						#21				#22				#23				#24				#25				#26				#27				#28				#29				#30							C
D						#31				#32				#33				#34				#35				#36				#37				#38				#39				#40							D
E						#41				#42				#43				#44				#45				#46				#47				#48				#49				#50							E
F						#51				#52				#53				#54				#55				#56				#57				#58				#59				#60							F
G						#61				#62				#63				#64				#65				#66				#67				#68				#69				#70							G
H						#71				#72				#73				#74				#75				#76				#77				#78				#79				#80							H
I						#81				#82				#83				#84				#85				#86				#87				#88				#89				#90							I
J						#91				#92				#93				#94				#95				#96				#97				#98				#99				#100							J
K	Empty		Control			#101				#102				#103				#104				#105				#106				#107				#108				#109				#110			Control		Empty		K
L						#111				#112				#113				#114				#115				#116				#117				#118				#119				#120							L
M						#121				#122				#123				#124				#125				#126				#127				#128				#129				#130							M
N						#131				#132				#133				#134				#135				#136				#137				#138				#139				#140							N
O						#141				#142				#143				#144				#145				#146				#147				#148				#149				#150							O
P						#151				#152				#153				#154				#155				#156				#157				#158				#159				#160							P
Q						#161				#162				#163				#164				#165				#166				#167				#168				#169				#170							Q
R						#171				#172				#173				#174				#175				#176				#177				#178				#179				#180							R
S						#181				#182				#183				#184				#185				#186				#187				#188				#189				#190							S
T						#191				#192				#193				#194				#195				#196				#197				#198				#199				#200							T
U						#201				#202				#203				#204				#205				#206				#207				#208				#209				#210							U
V						#211				#212				#213				#214				#215				#216				#217				#218				#219				#220							V
W						#221				#222				#223				#224				#225				#226				#227				#228				#229				#230							W
X						#231				#232				#233				#234				#235				#236				#237				#238				#239				#240							X
Y						#241				#242				#243				#244				#245				#246				#247				#248				#249				#250							Y
Z						#251				#252				#253				#254				#255				#256				#257				#258				#259				#260							Z
AA						#261				#262				#263				#264				#265				#266				#267				#268				#269				#270							AA
AB						#271				#272				#273				#274				#275				#276				#277				#278				#279				#280							AB
AC						#281				#282				#283				#284				#285				#286				#287				#288				#289				#290							AC
AD						#291				#292				#293				#294				#295				#296				#297				#298				#299				#300							AD
AE						#301				#302				#303				#304				#305				#306				#307				#308				#309				#310							AE
AF						#311				#312				#313				#314				#315				#316				#317				#318				#319				#320							AF
	1	2	3	4	5	6	7	8	9	10	11	12	13	14	15	16	17	18	19	20	21	22	23	24	25	26	27	28	29	30	31	32	33	34	35	36	37	38	39	40	41	42	43	44	45	46	47	48	
			D	D+A	D	D+A	D	D+A	D	D+A	D	D+A	D	D+A	D	D+A	D	D+A	D	D+A	D	D+A	D	D+A	D	D+A	D	D+A	D	D+A	D	D+A	D	D+A	D	D+A	D	D+A	D	D+A	D	D+A	D	D+A	D	D+A			

FRET FRET FRET FRET FRET FRET FRET FRET FRET FRET FRET FRET FRET FRET FRET FRET FRET FRET FRET FRET FRET FRET

2FRETs 2FRETs 2FRETs 2FRETs 2FRETs 2FRETs 2FRETs 2FRETs 2FRETs 2FRETs

Supplementary Figure S12. Experimental format of 1,536 well plates. 17 µm beads containing A455E D or D+A RNCs (acceptor 487 and truncation 654) were dispensed into odd or even number columns as indicated at bottom. Columns #1, #2, #47, and #48 were empty wells. Columns #3, #4, #45, and #46 were 0.1% DMSO control wells. 320 compounds (4 wells each compound) per plate were mixed with bead solution at final 10 uM concentration (0.1% DMSO) using columns #5-#44. Average of 2 FRETs was yielded from 2 paired D and D+A wells each compound as indicated at bottom.

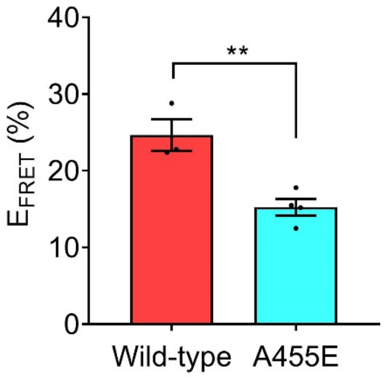

Supplementary Figure S13. Graph showing E_{FRET} values obtained from individual experiments of solid-support FRET assay for wild-type, or A455E constructs (acceptor 567 and truncation 674). (mean ± SEM, n=3-4 individual experiments). Two-tailed unpaired student's t-test, **p < 0.01.

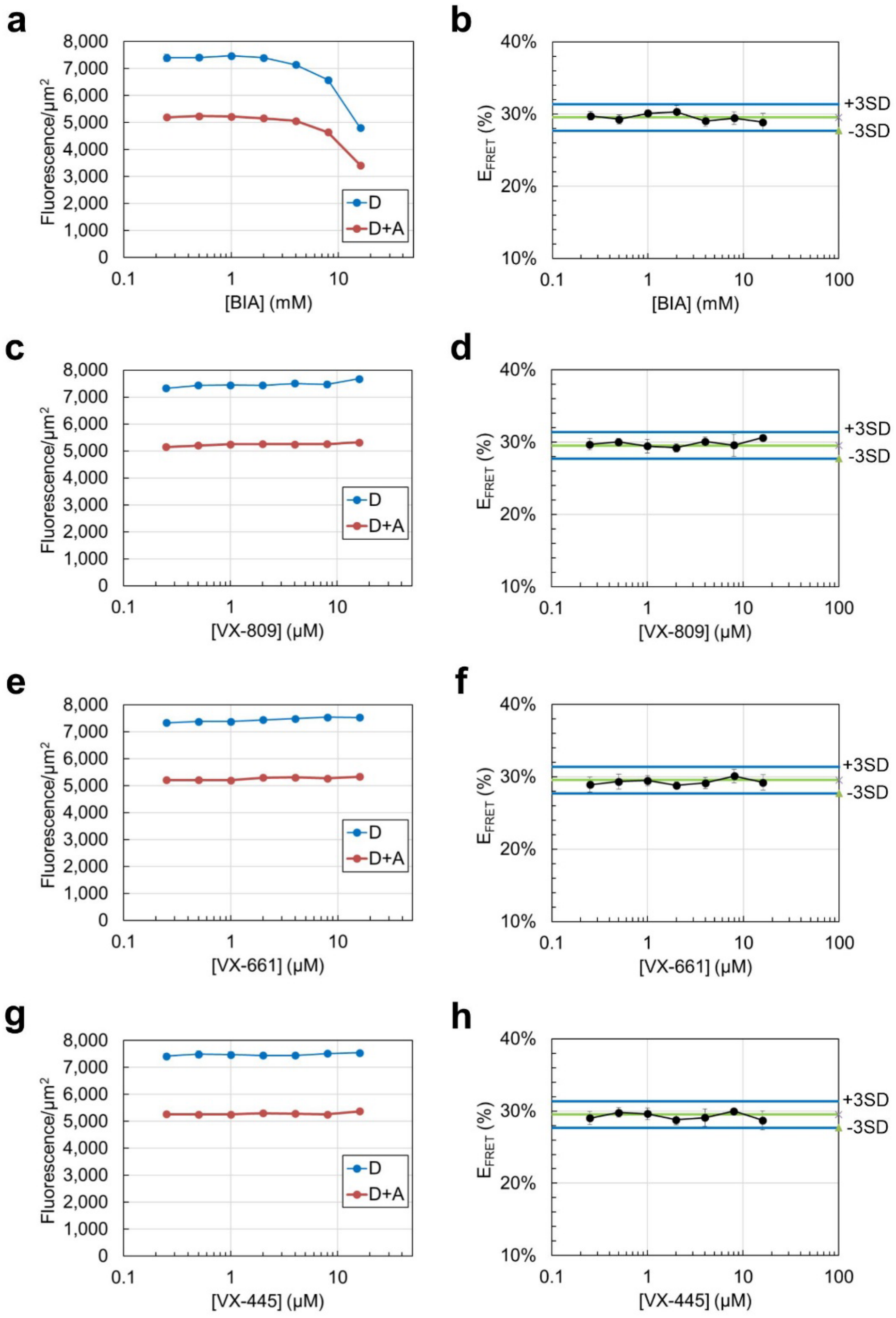

Supplementary Figure S14. Dose response results for an NBD1 binding small molecule and the Vertex correctors. Fluorescence intensities of beads containing bound D or D+A RNCs (mean ± SD) (**a, c, e, g**) and E_{FRET} (mean ± SD, n=3) (**b, d, f, h**) measured by solid-support FRET using A455E NBD1 RNCs with acceptor dye inserted at residue 487 for BIA (5-bromo-3-indole acetic acid) (**a**, **b**), VX-809 (**c**, **d**), VX-661 (**e**, **f**), or VX-445 (**g**, **h**).

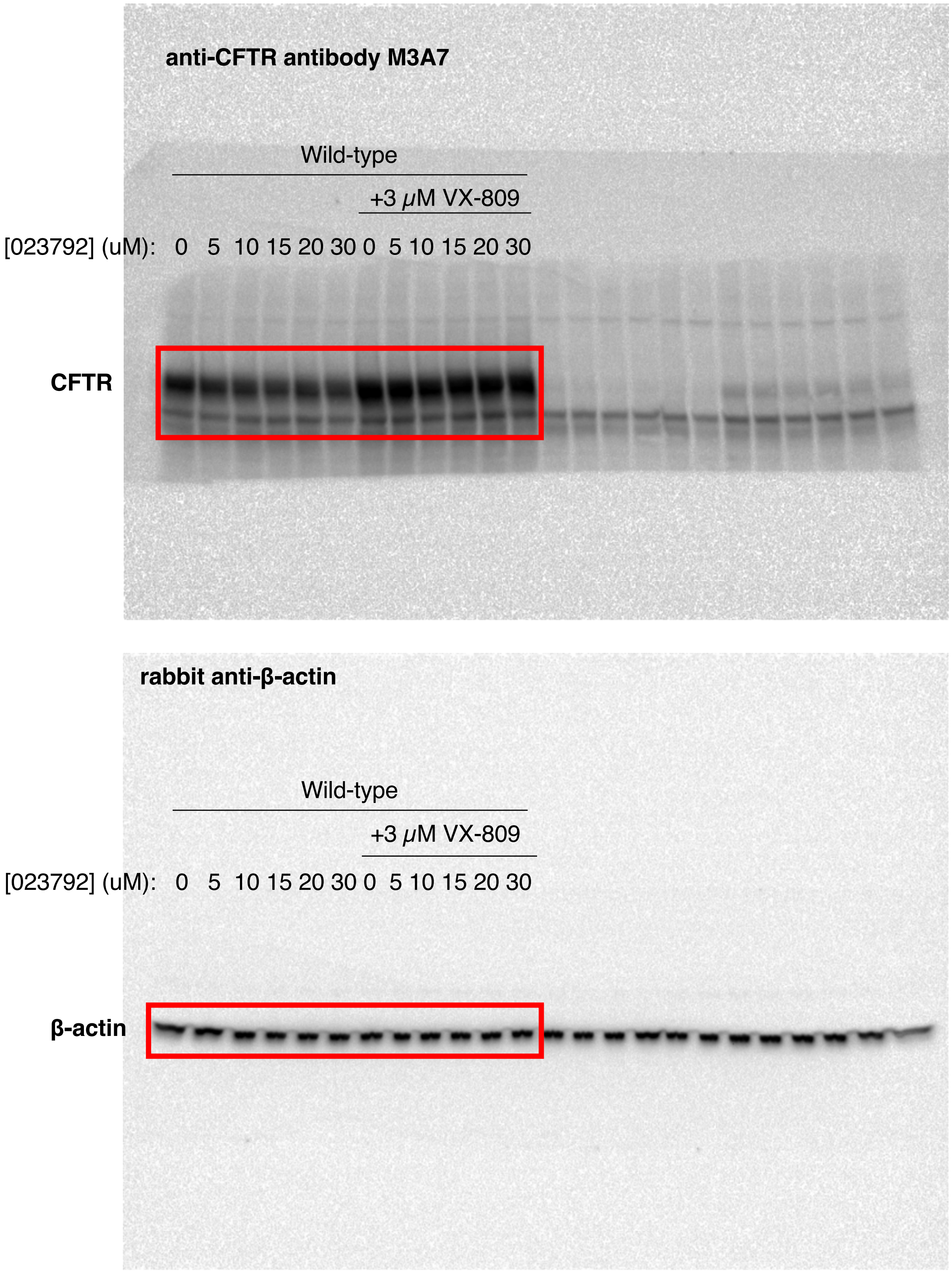

Supplementary Figure S15. Uncropped images of western blots in Fig. 8h. Molecular weight markers, visible protein standards, were used to cut relevant portion of gel for PVDF transfer. Standards were marked manually and were not visualized by HRP blotting method.

anti-CFTR antibody M3A7

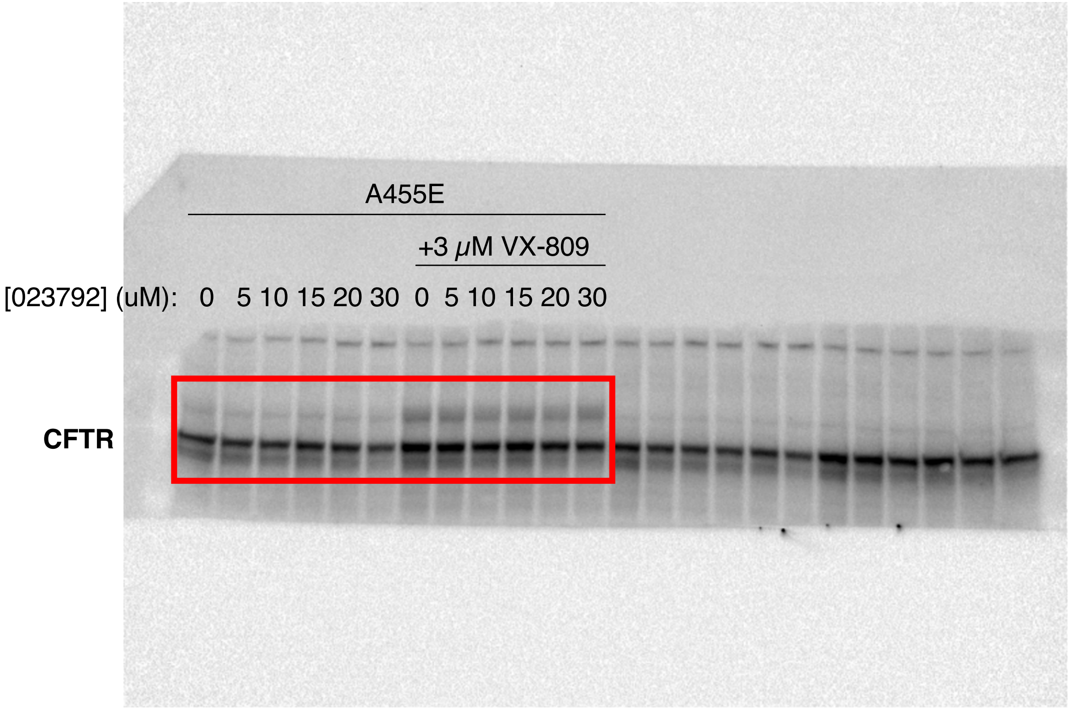

rabbit anti-β-actin

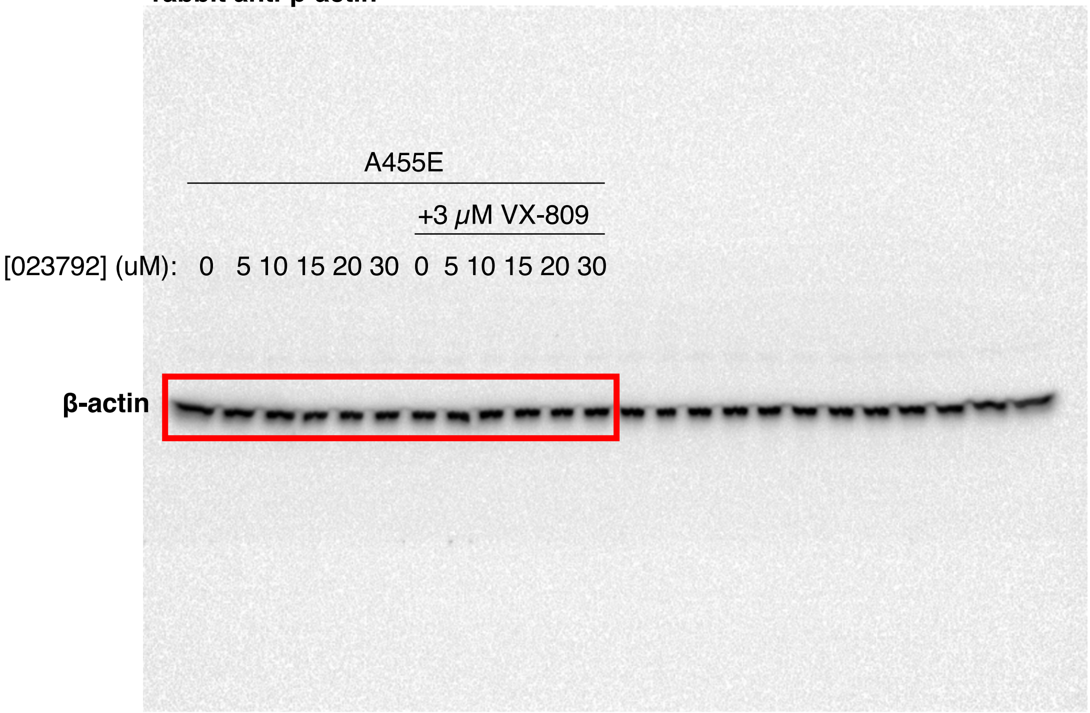

Supplementary Figure S16. Uncropped images of western blots in Fig. 8h. Molecular weight markers, visible protein standards, were used to cut relevant portion of gel for PVDF transfer. Standards were marked manually and were not visualized by HRP blotting method.

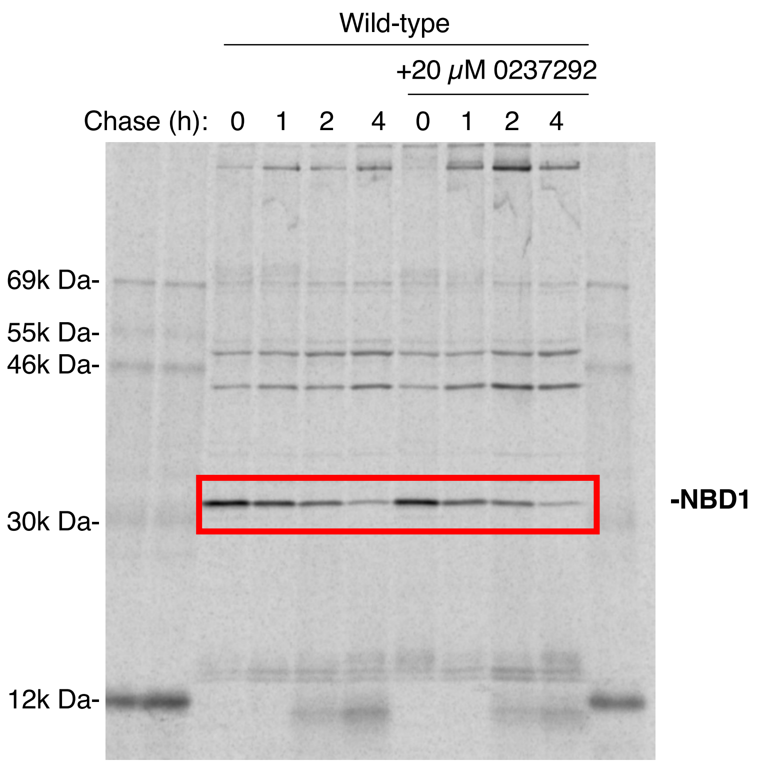

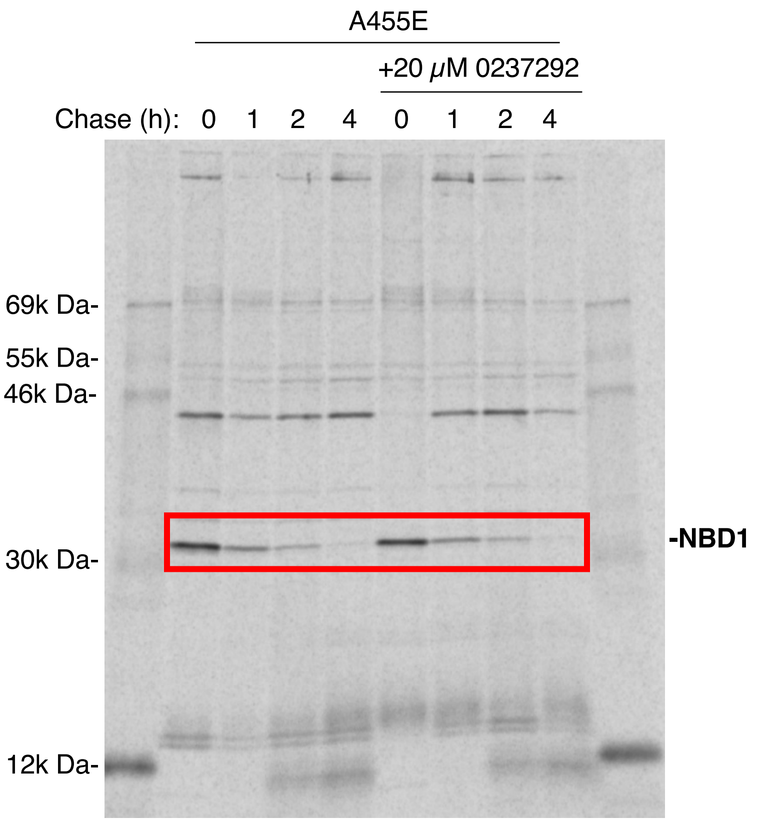

Supplementary Figure S17. Uncropped images of SDS-PAGE gels in Fig. 8i.

Supplementary Table S1. Summary of beads tested for RNC binding. Summary of solid supports tested for RNC binding showing various tags used and general results.

	Product Name	Manufacturer	Size	Binding Capacity (/ μm^2)	Target	Autofluorescence	Net Signal
1	Streptavidin Agarose resin	Thermo	45-165 µm	2.0×10^5 molecules	SBP-tag	Low	+
2	TALON Metal Affinity Resin CL-6B (Cobalt)	Clontech	45-165 µm	1.2×10^6 molecules	His-tag	Low	+++
3	Ni-NTA Agarose	QIAGEN	45-165 µm	3.5×10^6 molecules	His-tag	Low	+++++
4	HiTrap Chelating HP	GE Healthcare	34µm	8.4×10^5 molecules	His-tag	Low	+++++
5	High Density Nickel 4 Highly Cross-linked Superfine 17µm	Agarose Bead Technologies	17 µm	Not provided	His-tag	Low	+++++
6	Dynabeads His-tag isolation & pulldown	Life Tech.	1 µm	2.5×10^7 molecules	His-tag	High	+++++
7	PureProteom Nickel Magnetic Beads	Millipore	10 µm	2.4×10^6 molecules	His-tag	Low	++
8	TALON® Magnetic Beads	Clontech	25-75 µm	Not provided	His-tag	Low	++
9	Affigel Protein A	Bio-rad	Not provided	Not provided	Myc-tag via antibody	Low	+
10	Protein A Magnetic Beads	New England Biolabs	10 µm	Not provided	Myc-tag via antibody	High	ND
11	Mouse IgG Coated Polystyrene Particles	Spherotech	5 µm	Not provided	Myc-tag via antibody	High	ND
12	Protein A Coated Polystyrene Particles	Spherotech	5 µm	Not provided	Myc-tag via antibody	High	ND
